# Ten Years Progress of Electrical Detection of Heavy Metal Ions (HMIs) Using Various Field-Effect Transistor (FET) Nanosensors: A Review

**DOI:** 10.3390/bios11120478

**Published:** 2021-11-25

**Authors:** Shaili Falina, Mohd Syamsul, Nuha Abd Rhaffor, Sofiyah Sal Hamid, Khairu Anuar Mohamed Zain, Asrulnizam Abd Manaf, Hiroshi Kawarada

**Affiliations:** 1 Collaborative Microelectronic Design Excellence Center (CEDEC), Universiti Sains Malaysia, Sains@USM, Bayan Lepas 11900, Pulau Pinang, Malaysia; shailifalina@moegi.waseda.jp (S.F.); nuha@usm.my (N.A.R.); sofiyah@usm.my (S.S.H.); anuar@usm.my (K.A.M.Z.); 2 Faculty of Science and Engineering, Waseda University, Tokyo 169-8555, Japan; kawarada@waseda.jp; 3 Institute of Nano Optoelectronics Research and Technology (INOR), Universiti Sains Malaysia, Sains@USM, Bayan Lepas 11900, Pulau Pinang, Malaysia; 4 The Kagami Memorial Laboratory for Materials Science and Technology, Waseda University, 2-8-26 Nishiwaseda, Shinjuku, Tokyo 169-0051, Japan

**Keywords:** heavy metal ions, field-effect transistor, HEMT, chemiresistor, electrical detection, nanomaterials, Debye length, screening effect

## Abstract

Heavy metal pollution remains a major concern for the public today, in line with the growing population and global industrialization. Heavy metal ion (HMI) is a threat to human and environmental safety, even at low concentrations, thus rapid and continuous HMI monitoring is essential. Among the sensors available for HMI detection, the field-effect transistor (FET) sensor demonstrates promising potential for fast and real-time detection. The aim of this review is to provide a condensed overview of the contribution of certain semiconductor substrates in the development of chemical and biosensor FETs for HMI detection in the past decade. A brief introduction of the FET sensor along with its construction and configuration is presented in the first part of this review. Subsequently, the FET sensor deployment issue and FET intrinsic limitation screening effect are also discussed, and the solutions to overcome these shortcomings are summarized. Later, we summarize the strategies for HMIs’ electrical detection, mechanisms, and sensing performance on nanomaterial semiconductor FET transducers, including silicon, carbon nanotubes, graphene, AlGaN/GaN, transition metal dichalcogenides (TMD), black phosphorus, organic and inorganic semiconductor. Finally, concerns and suggestions regarding detection in the real samples using FET sensors are highlighted in the conclusion.

## 1. Introduction

Heavy metals are naturally occurring elements, produced from natural sources such as volcanic eruption, rock weathering, metal corrosion and metal evaporation from soil and water. Increasing human anthropogenic activities, including mining, industrial, agriculture and metallurgical, have escalated the heavy metal ions (HMIs) accumulation in our environment. The HMIs from these sources accumulate in the air, in drinking water, on plants, in animals, on soil, and on the earth’s surface. Consumption of these polluted plants, animals and drinking water can transfer the HMIs to humans, resulting in bioaccumulation. The toxicity of HMIs has a greater impact on children than on adults. A higher dose of HMIs among children may induce organ damage and promote neurotoxicity, which may cause behavioral disorders, learning problems and impaired growth in children’s development [1]. The Environmental Quality Standards Directive List has registered arsenic (As), cadmium (Cd), copper (Cu), chromium (Cr), iron (Fe), lead (Pb), mercury (Hg), nickel (Ni) and zinc (Zn) as prime substances of concern in water quality [2]. HMIs are imperative for life in trace quantity; however, they are hazardous to human health at higher concentrations. Real-time and constant HMIs monitoring is thus critical, reaching low concentrations (<nM). Table 1 summarizes the limit value, toxicity and heavy metal sources for heavy metal ions.

**Figure 1:**
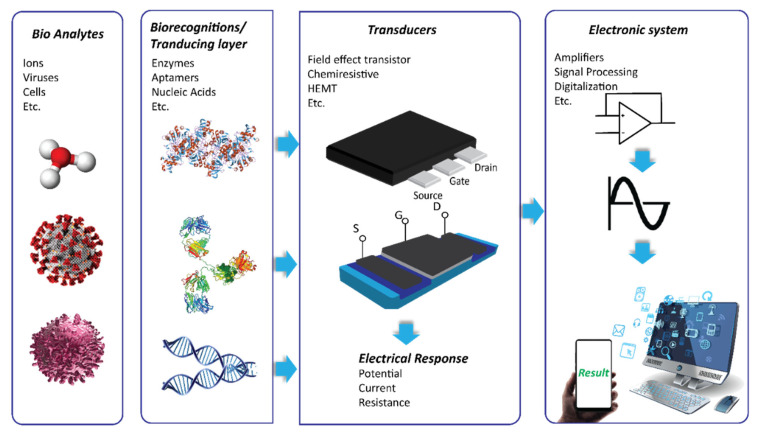
Scheme of a chemical and biosensor comprising of recognition element (receptor) on transducer to selectively recognize target of interest. The transducer changes the biorecognition event on its surface into a measurable signal before sending it for signal processing, which can be read by the end-user.

FET chemical sensor and biosensor systems for HMI detection consist of different functional blocks to create and measure analyte-specific signals, as presented in Figure 1. In this system, the recognition element (such as antibody, single-stranded DNA, ion-sensing membrane, etc.) and the transducer are fundamental components. The recognition element enables the binding of the probes-target to generate a signal, which corresponds to the presence of the HMIs and its abundance. In the case of biosensors, the recognition element is often conjugated on a solid surface layer on the transducer by immobilization. A transducer is a material that is responsible for converting the quantitative or semi-quantitative information about the target into a measurable signal (e.g., current, potential, and temperature change). Graphene, carbon nanotubes and silicon are the popular choice as a nanosensor transducer. The potential of these semiconductor and a few other semiconductor materials as transducers in FET/chemiresistive sensor development will be discussed in detail, and their application in HMIs’ electrical detection approach is critically reviewed. To date, different transducer platforms have been used to develop chemical or biosensors for HMIs monitoring, and a significant number of recognition elements has been explored to realize a wide range of HMIs detection.

Conventional detection methods, including atomic absorption spectroscopy (AAS) [4], inductively coupled plasma-atomic emission spectroscopy (ICP-AES) [5] and X-RAY fluorescence [6], are often used to conduct HMIs monitoring. However, these detection methods mostly require bulky equipment and trained personnel to perform the analysis. Recent developments in the field of sensors have led to a renewed interest in the miniaturization of three electrode-based electrochemical and an optical biosensor [7,8,9,10,11]. The newer sensor technology certainly offers the portability of the sensor and is more convenient to use. Nevertheless, most of the three electrode-based electrochemical and fluorescent sensors (regardless of size), require additional reagents for sensing operation, which limits their application as standard and simple analytical tools. Three-electrode electrochemical sensors require a redox mediator to assist the electrochemical activity on the electrode surface. It should be noted that the selection of a mediator could largely affect the sensitivity and selectivity of the electrochemical sensor. A previous study reported that the mediator may react with the heavy metal ions during the assessment, consequently influencing the accuracy of the electrochemical sensor [12]. In the case of a fluorescent biosensors, its sensing operation often requires pre-labelling the target with fluorescent dye [13]. This pre-labelling step in the fluorescent sensor complicates the assay procedure. When compared to electrochemical and fluorescent biosensors, FET biosensors assure rapid and accurate detection without the needs for additional reagents in their analytical assay. Therefore, the FET biosensor has the advantage of simpler handling for on-site application.

There are several types of commercially available HMI sensors on the market, including color test strips and color reagent kits (https://www.simplexhealth.co.uk/product/heavy-metals-test-kit-presence-1-test/, accessed on 16 November 2021) [14]. Both test kits change color with the presence of certain heavy metal ions. Even though it is considered cheap and convenient, there are several drawbacks associated with this kind of detection. Firstly, the test result reading is based on the color chart provided by the manufacturer, which can sometimes be misread by the user. Secondly, the reagent color only changes with the presence of heavy metal ions above µM (Cd = 44.80 µM, Cu = 78.69 µM, Pb = 24.13 µM, Hg = 24.93 µM, Ni = 85.19 µM, Zn = 76.45 µM, etc.) [14]. Finally, this colorimetric test cannot perform multiplex sensing, where the color reagent will turn grey in the presence of more than one species of HMIs at the same time. For these reasons, electrical detection, such as FET-based sensors, appears to be the most promising alternative to existing commercial HMIs sensors. FET offers rapid detection and accurate test results. Moreover, with appropriate receptors, the FET sensor allows for detection of HMIs at levels below nanomolar (nM). Multiple HMI detection is very possible with FET array sensors.

The past 50 years have seen increasingly rapid advances in the field of FET-based sensing since it was first introduced by P. Bergveld [15] for measuring ions’ activity in an electrolyte in a short communication dating back to the 1970s. The ion-sensitive FET (ISFET) derived from the metal-oxide-semiconductor FET (MOSFET) with the metal gate was removed and the gate terminal of the ISFET was governed by a reference electrode inserted into an aqueous solution, which is in contact with the underlying metal gate oxide. The earliest research on ISFET focused on monitoring ion concentration in a solution [16,17,18]; however, 12 years later, ISFET has been employed as a biosensor. For example, Janata et al. developed immunoFET for detection of anti-syphilis and anti-albumin antibodies [19]. To date, a vast body of literature has reported the work on ISFET with various types of gate-modification techniques, electrode materials, and sensing membranes. Not limited to biomolecular sensing, considerable efforts have been made to develop ISFETs for HMIs detection. Due to changes in gate modification or sensing membranes, ISFETs evolved into a variety of shapes and sizes, including the solution-gate field effect transistor (SGFET), chemical sensor FET (Chem-FET) and extended gate FET. The developments in the field of semiconductor fabrication have led to a renewed interest in FET-based sensors.

This review aims to provide an overview of Chem/BioFET sensor studies that have been dedicated to HMI detection for the past 10 years (from 2010 to present). To best of our knowledge, there are many reviews on the development of electrochemical [20,21,22], voltammetry [23,24,25] and fluorescence [26,27] HMI sensor; however, the review specifically on the electrical detection of HMIs is scarcely available in the literature. This review has been prepared in a way that comprehensively covers the information about all the aspects of the Chem/BioFET sensor, which could be useful to provide insights for researchers and scientists to accelerate their innovation for the detection of HMIs. This review begins with an introduction to FET, followed by the configuration and general FET setup. The FET intrinsic issues (screening effect and the Debye length) and their solutions to overcome these shortcomings are discussed in the next section, besides addressing the challenges of the FET sensor deployment. The following section will discuss the development of FET for HMIs sensing based on the types of semiconductors (silicon, carbon, III-V compound, transition metal dichalcogenide (TMD), and other materials, such as black phosphorus, organic-based FET and inorganic-based FET). Finally, the current challenge of using FET sensors for detection in real samples and suggestions to address the issue will be highlighted.

## 2. FET Configurations and General Experiment Setup for Electrical Detection of Heavy Metal Ions

Electrical detection of HMIs involves the use of FETs to generate a measurable electrical signal corresponding to the interaction of HMIs on the FET channel sensing interface. When the HMI bounds onto the channel area and creates an ionic bond with the sensing material, the surface potential changes, subsequently inducing charge accumulation near the semiconductor/analyte interface, thus modulates the carrier density in the channel area. For example, the negative ions on the sensing surface of an n-channel FET repel the electron carriers in the channel, resulting in a decrease of drain current, thus reflecting a low output signal. However, positive ions that are bound on the sensing surface will attract more electrons in the channel area, so there will be an increase in drain current. While explaining the detection mechanism of an FET in this manner appears to be straightforward, in practice, a few other parameters are involved, including the screening effect, the Debye length and the gate capacitance discussed in Section 3.

**Figure 2:**
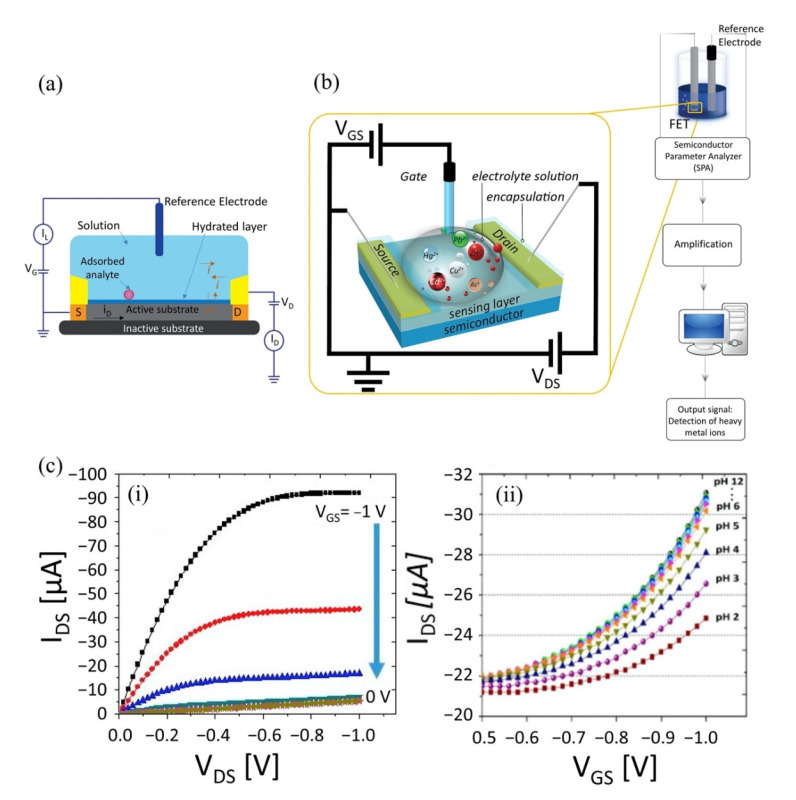
Schematic of (**a**) cross-section of FET sensor and (**b**) general equipment setup for HMIs electrical detection in laboratory. (**c**-**i**) An example of the current-voltage characteristics of a diamond-based SGFET and (**c**-**ii**) changes in cur-rent-voltage characteristics of diamond-based SGFET in a wide range of pH solutions (pH 2–pH 12). Reproduced with permission from [34]. Copyright 2018 MDPI.

A typical FET configuration comprises three terminals: source, drain and gate, as shown in Figure 2a. The source and drain are symmetrical metal pads separated by a narrow channel area. In the FET setup, a voltage is applied at the drain (D) to enable current flow from the source to the drain terminal. While a gate voltage is applied at the gate, terminal (G) produces field-effect to control the current flow, and the source (S) terminal is usually connected to the ground. For HMI detection, the FET sensor is designed to adapt to the aqueous environment as most detection is carried out in solution. The FET that operates in an aqueous/electrolyte environment is customarily known as a solution-gate field-effect transistor (SGFET) or an electrolyte-gate field-effect transistor (EGFET). Both the source and drain of the SGFET/EGFET are passivated with waterproof materials to prevent ions from the analyte penetrating the circuit, while the metal gate electrode is replaced with a silver chloride (AgCl/Cl) reference electrode to control the field-effect between the source and drain terminals. The channel area is modified with a specific sensing interface or probe, such as nanoparticles, deoxrybozyme (DNAzymes), proteins, and aptamers for detection purposes. The selectivity and sensitivity performance of the SGFET/EGFET sensor is determined by the sensing interfaces. A typical FET sensor device requires gate dielectric layers to avoid unstable potential and impedance at the semiconductor/analyte interface. Earlier, silicon dioxide (SiO_2_) was a popular material used for the gate dielectric and semiconductor/liquid interface because of its stability when in contact with the electrolyte [28]. However, researchers eventually replaced SiO_2_ with other oxides (Si_3_N_4_, Al_2_O_3_, or Ta_2_O_5_) due to drift issues [29]. It is worth noting that some of the FET sensors, such as graphene SGFET [30,31] and diamond SGFET [32], allow for direct detection without requiring a gate dielectric or insulating interface. In such cases, the recognition probes are affixed directly on the semiconductor surface.

The electrical detection of HMIs in the laboratory is illustrated in Figure 2b. The setup consists of a SGFET sensor immersed in an analyte that contains HMIs, the AgCl/Cl reference electrode is used to supply gate voltage (V_G_), and external voltages to supply the excitation signal at the drain and gate terminals simultaneously to measure the sensor response. A semiconductor device parameter analyzer is used for this purpose. Voltages at the drain and gate terminals are varied, depending on the respective semiconductor material potential window, whereas the source terminal is kept constant (grounded). The electrical signal response upon detection of HMIs can be observed with the changes in the current-voltage characteristics of the SGFET sensor. Figure 2c(i) is an example of the current-voltage characteristics of a SGFET with p-type behavior, measured with gate voltage (V_GS_) values from 0 V to 1 V with 0.2 V of step (ΔV_GS_), while in Figure 2c(ii) is an example of changes in current-voltage characteristics of the SGFET sensor at different pH values (pH 2 to pH 12). HMI detection in a significantly low analyte concentration results in weak signals, hence amplification is needed prior to sending the response signal to any handheld device reader to interpret and analyze the recognition event. The amplification strategy is imperative to improve the signal-to-noise ratio (SNR) to detect target analytes in the case of high extrinsic noise. The silicon nanowire FET biosensor with rolling circle amplification (RCA) strategy exhibited an impressive signal-to-noise ratio (SNR) of >20, which could simplify the demand on the readout system [33].

## 3. FET Sensor Challenges

### 3.1. Screening Effect and Debye Length

Over the past decade, there has been exciting progress in FET sensor development. Researchers report various FET sensing interface designs that can specifically bind to the target of interest [35,36]. However, the sensitivity of the FET sensor is limited by the ionic screening effect, which remains a challenge, especially in high ionic concentration solutions (10× phosphate buffered saline) or physiological conditions. When a semiconductor solid surface is in contact with an electrolyte, some of the free mobile ions in the electrolyte approach the semiconductor surface and rearrange themselves as one layer of screening ions, creating an electric double layer, E_DL_. At a Debye length of ~1 nm (comparable to 100 mM in ionic concentration), the target charges can be electrically detected on the sensing surface [37]. However, when a captured target charge is located at a distance further than the Debye length, the electrostatic potential is shielded by these screening ions; therefore, the FET sensing surface could not detect this binding event. Moreover, the Gouy–Chapman–Stern diffuse double layer model advocates that the mismatch in size between Debye length and the target analytes’ charge would hinder the detection at the sensing surface [38]. Generally, in physiological conditions and high concentrated solutions, the Debye length appears to be short (~0.7–1 nm) [39]. Whereas most of the target charges are in medium-sized and large-sized molecules (the size radius can be up to 10 nm). Both circumstances contribute to a challenge in FET real-time detection in clinical samples. This section will discuss the Debye screening length and approaches that have been explored to overcome this limitation on the FET sensor. We note that the approaches presented in this review are not limited only to HMIs detection, but also include methods and strategies to overcome screening effects in general.

**Figure 3:**
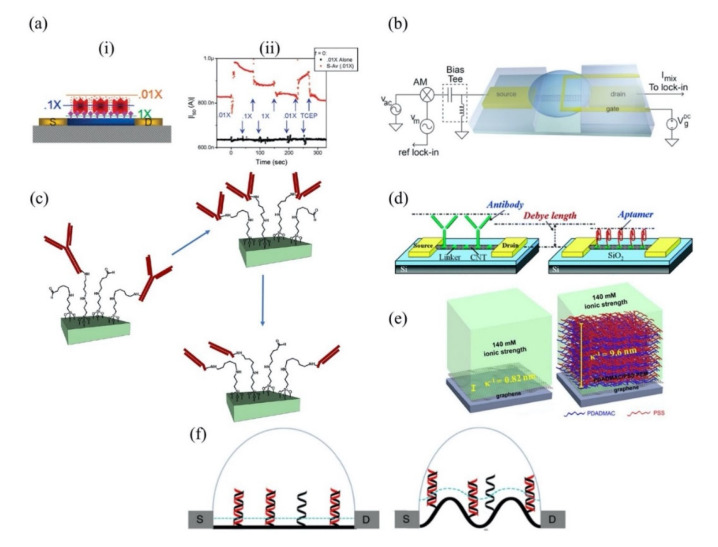
FET biosensors methodologies to overcome screening limitation (**a**-**i**) Debye length (λ_D_) model in different ionic strength by Stern et al. The Debye length in 1 × PBS, 0.1 × PBS and 0.01 × PBS represent by the green line, blue line and orange line, respectively. (**a**-**ii**) The response of the biotin-functionalized FET for streptavidin detection in different ionic strength. Reprinted (adapted) with permission from [41]. Copyright 2007 American Chemical Society. (**b**) Geometry of a FET sensor device with mixing current measurement setup for high-frequency sensor device operation. Reprinted (adapted) with permission from [42]. Copyright 2012 American Chemical Society. (**c**) Generation of antibody fragments from a whole of antibody molecule as proposed by Elnathan. Reprinted (adapted) with permission from [43]. Copyright 2012 American Chemical Society. (**d**) Size comparison between aptamer and antibody receptor. The aptamer is smaller and fits well within the Debye length, resulting in higher sensitivity detection compared to other bioreceptors, such antibody. Reprinted (adapted) with permission from [44]. Copyright 2007 American Chemical Society. (**e**) Construction of PDADMAC/PSS polyelectrolyte multilayer (PEM) films on graphene FET. Reprinted (adapted) with permission from [45]. Copyright 2018 American Chemical Society. (**f**) Comparison of Debye length represented by blue dot line on flat graphene surface (left) and on crumpled graphene surface (right). Debye length increased at the convex region of crumpled graphene surface. Probe and target DNA represented in black and red, respectively. Reprint with permission from reference [47]. Copyright 2020 Springer Nature.

The sensitivity of a FET is strongly correlated with the Debye length. Certainly, the Debye length is an important parameter to be considered when designing a FET sensor. Many efforts have been made over recent years to overcome this FET shortcoming. A common technique to reduce screening effects is by diluting the test sample with buffer solution to reduce ion concentration [40]. A study by Stern et al. demonstrated the sensitivity of the FET sensor is significantly improved in low-concentrated electrolyte compared to a high ionic strength solution [41]. Figure 3a(i) elucidates the relationship between Debye length and ionic strength according to Stern et al. A 10-fold decrease in ionic strength extends Debye length from the device surface. Biotin-functionalized FET sensor for the detection of streptavidin has confirmed that the dilution of an electrolyte affects the Debye length. The sensor response depends on ionic strength resulting from different Debye lengths, as shown in Figure 3a(ii). While this method is effective for minimizing the screening effect, it also affects the binding of ligand–protein and protein–protein interactions. Therefore, this method is impractical for real-time detection. Kulkarni et al. exploited high transconductance silicon nanowires (SiNWs) to operate the FET sensor at high frequency [42]. This method presents outstanding sensor performance in highly concentrated solutions up to 100 mM. However, the complex sensor device geometry complicates its application in cellular or vivo sensing, as shown in Figure 3b. A study by Elnathan et al. [43] found that the fragmentation of biorecognition probes can improve the sensitivity of FET sensors in physiological conditions such as serum and blood without sample pre-treatment. In the study, Elnathan disintegrated antigen binding from antibodies for detection of proteins to reduce the size of the sensing probe, so that it is closer to the sensing surface and falls within the Debye screening length for high sensitivity detection. Figure 3c illustrates two steps in which the antibody degrades into fragments to reduce the antibody receptor size as proposed by Elnathan. One way to diminish the screen effect is the use of aptamers as biorecognition probes. Because the size of the aptamer is smaller than the Debye length (Figure 3d), this allows for binding events to occur within the electrical double layer and yields a high sensitivity sensor response. On this basis, Maehashi et al. developed an FET sensor functionalized with aptamer for immunoglobulin E (IgE) detection [44]. In another report, the use of polyelectrolyte layers effectively increased the sensing range, as depicted in Figure 3e. Paccinini et al. introduced PDADMAC/PSS polyelectrolyte multilayer (PEM) films on the graphene FET sensor to increase the sensing range [45]. Interestingly, the Debye length was found to increase from 0.8 to 10 nm, enabling detection of bigger biomolecules. Furthermore, Lieber et al. suggested the use of a biomolecule-permeable polyethylene glycol (PEG) polymer layer on the SiNWs FET sensing area to extend the Debye length in high ionic strength solution [46]. They showed the FET sensor device with PEG-modified exhibits real-time detection of prostate specific antigen (PSA) in concentrations as high as 150 mM. To validate their hypothesis, the outcome was compared to the performance of the FET sensor without PEG-modified as the control, and it was found the control only enables detection of PSA in concentration ≤10 nM. In this study, it was proven that the FET sensor with a PEG layer is effective for PSA detection and demonstrates real-time detection in a 1× PBS solution, which mimics the physiological environment. A recent study showed that the screening effect of an FET can be reduced using rippled sensing area (channel). The study particularly demonstrated the crumpled (deformed and bent) graphene FET biosensor for ultrasensitive detection of DNA/RNA molecules [47]. The crumpled form of graphene was attained by manipulation of the two-dimensional (2-D) layer with controlled heat to induce deformation of the underlying substrate. With a crumpled sensing surface, the Debye length fluctuated at the peaks and the valleys, as shown in Figure 3f. The performance of the crumpled graphene FET as a biosensor was compared with that of the flat graphene FET, which was used as a negative control. The first set of analyses examined crumpled graphene FET responses in different pH solutions. Compared to the control group, the crumpled graphene FET demonstrated a larger shift in Dirac point from pH 3 to pH 11. Turning now to the experimental evidence on DNA sensing of crumpled graphene FET in DNA solution, the flat graphene FET showed a total Dirac point shift of 80 mV, whereas the crumpled graphene FET exhibited a total Dirac point shift up to 180 mV. They further evaluated the performance of the crumpled graphene FET sensor in a clinical sample (undiluted human serum) to detect miRNA. Even in high ionic and complex matrices of biological components, the sensor demonstrated excellent potential as a highly sensitive biosensor, which showed detection of miRNA molecules in the human serum as low as 20 aM of concentration. These experimental data have supported the hypothesis that the screening limitation of a graphene FET can be eliminated by using crumpled graphene on the sensing area. Their computational simulation data revealed that the fluctuated area (peaks and valleys of crumpled graphene) had higher DNA adsorption energies, especially on the concave area (−532.187 kcal mol^−1^). They deduced that the extraordinary performance of the crumpled graphene FET sensor was contributed by these valleys’ regions (concave areas). Due to the nature of a concave region with deep and narrow trenches, the free mobile ions in the tested solution were excluded and farther away, which allowed the DNA to get closer to the graphene surface, thereby inducing more graphene carrier density, resulting in a larger Dirac point shift. However, this method is only applicable to 2-D materials or thin films that can create ripple effects.

### 3.2. Sensor Deployment

Sensor portability is a popular solution to conventional benchtop laboratory sensors for ease of implementation and cost-effectiveness. Recent advancements in nanoscale devices have led to the miniaturization of biosensors, which show promising potential to be deployed as self-contained lab-on-chip devices. While the research of nanosensors in the laboratory shows significant sensing performance, several important challenges remain to be addressed before the portable sensors can be deployed on a large scale. In this review, the focus is on three challenges to deploy FET sensors, which are in terms of selectivity, platform integration/reading and real-time measurement.

The important aspects concerning a sensor are sensitivity and selectivity, which are determined by the recognition elements or receptors on the sensing surface. An appropriate sensor receptor must be carefully chosen to obtain a high sensitivity sensor. With regard to selectivity, the recognition elements often show cross-selectivity with interference ions in complex matrices. In general, the lower the detection limit is required, the more difficult for a sensor to identify a specific target in the given matrix, so the sensor may give misleading (false positive) results. This is an important issue that needs to be addressed, especially for self-operating system sensors. One possible solution is to fabricate the sensors in an array for multiplex detection, in which each sensor is functionalized with different receptors. The sensor signal would contain a vector of responses, which is treated as a detection pattern by the end users. In this way, false results can be avoided when the samples of complex matrices are assayed. The conventional approach to realizing multiplex detection is to fabricate a multiple-channel FET either with a one-shared gate terminal or an individual side gate terminal on each channel to allow individual access to each FET [48]. In this approach to sensing multiple target ions, each FET channel is immobilized with different biorecognition molecules. A proof-of-concept study demonstrated multiplex detection of prostate specific antigen (PSA) and PSA-α1-antichymotrypsin (PSA-ACT) with a silicon nanowire FET array [49]. The individual FET sensor devices were modified with different monoclonal antibodies (mAbs) that corresponded to the target proteins. The first FET (NW1) was functionalized with mAbs for free PSA (Ab1). While the second FET (NW2) was functionalized with cross-react mAbs of free PSA and PSA-ACT (Ab2). This study found that the FET conductance changes were observed in both NW1 and NW2 when free PSA was introduced to the sensor. The introduction of PSA-ACT yielded conductance changes only in NW2, suggesting specific binding of PSA-ACT on the sensor device NW2. These findings suggest that the FET sensor array has the potential for multiplex detection of free PSA and PSA-ACT proteins at the same time. The study also investigated the selective detection of either cancer biomarker in a single real-time assay, with the help of an additional blocking solution to block off the other target. For example, a solution of Ab1 was mixed into the mixture of free PSA and PSA-ACT solution before being introduced to the sensor array to block off the free PSA target, resulting in conductance changes in NW2, but not in NW1. 

Most single chemical- and biosensors (including FET) exhibit limitations in multiple detections in complex matrices. As demonstrated above, they can only be used to determine one or two types of analytes simultaneously. Therefore, a multisensory system is needed to overcome this challenge. Such a sensor system has been widely studied and recognized as electronic tongue (e-tongue) [50]. Generally, e-tongue was developed to mimic human chemical senses for liquid analysis, especially for food and beverages. However, its application to date has extended to biology, pharmaceutical and environmental fields. The e-tongue is comprised of poorly selective and lower-specificity chemical sensor arrays coupled with pattern recognition math methods (PARCs) to analyze multiple target analytes simultaneously. Common e-tongue pattern recognition uses chemometric tools, which have been used to translate hundreds or thousands of signal data onto visualization maps [51]. E-tongue is a promising low-cost detection system, and it can be integrated into the internet of things (IoT). Additionally, the combination of e-tongue with a pattern recognition algorithm is a powerful analytical tool for the rapid and accurate detection of ions in complex media. From our perspective, the performance of e-tongue cannot be compared to that of a FET biosensor, as e-tongue is a complete sensor system, while FET performance is often evaluated at a single device level. However, a FET biosensor can be incorporated into an e-tongue system to achieve rapid and accurate multiple detection. Several studies attempted to develop FET ion sensors towards e-tongue applications [52,53,54]. The e-tongue for heavy metal ions detection has been comprehensively discussed elsewhere [55].

Detection of low abundance of HMI in a real sample can be divided into two steps: (1) separation of high-abundant non-specific target molecules and (2) increasing efficiency of mass transfer target (HMI) to the sensing area. A common problem faced by a biosensor assay in a raw sample is being unable to distinguish between target and non-target molecules. Therefore, the target molecule needs to be isolated from non-specific molecules before being analyzed by a biosensor. It has been demonstrated that the detection of raw samples that undergo a separation step prior to introduction to a biosensing surface is more efficient [56]. The separation of the target molecules can be realized with different tools/techniques, such as micromixing [57], immunomagnetic beads [58], dielectrophoresis (DEP) [59], filtration [60] and centrifugation [61]. Among these separation methods/techniques, centrifugation is popular for isolating target molecules due to its simplicity and efficiency compared to the other methods. Although centrifugation could not separate the HMI-contaminated solution into different ion species; however, it could aid in the separation of HMI from complex water matrices [62,63,64,65]. One significant advantage of the centrifugation step in the HMI detection assay is that it discards impurities and interfering non-target species on the sensor surface, thereby promoting better selectivity of the sensor. Moving on to look at DEP implementation in biosensors to improve the sensitivity and selectivity of the biosensor assay. In brief, DEP is a non-uniform electric field that is applied to the biological fluid to manipulate the position of the biomolecules in a given region. The inhomogeneity of DEP imposes a net force on biomolecules and pushes them towards/off the electric field region depending upon the polarization between biomolecules and the suspending medium. The DEP force is particle size dependent, which helps to separate molecules into different regions based on their type. This technique is particularly useful for concentrating the target sample on the recognition probe to enhance the sensitivity of a biosensor [66]. DEP can also be used to filtrate and purify the biology target samples [67], to discriminate between specific and non-specific bindings of biology components [68], and to deposit biological targets onto desired the location on sensing platform of field-effect based biosensor [69]. In FET application, one study reported DEP for pre-concentrated deoxyribonucleic acid (DNA) analyte on poly-silicon nanowire field-effect transistor (poly-Si NWFET) [70]. In this study, a three-dimensional (3-D) microstructure of poly (ethylene glycol) diacrylate (PEG-DA) was used to shape the electric field on the micro-constricted fluid channel onto the poly-Si NWFET sensing region. The DNA pre-concentration degree increased by 4-fold, 5-fold, and 4-fold in the constriction channels of 6, 10 and 20 µm, respectively. The gate voltage of the poly-Si NWFET sensor shifted by 0.5 V when a DEP condition of 30 Vpp (peak to peak) at 500 Hz was applied for 5 min to a double stranded DNA analyte (20 bp) of 1 nM concentration in sodium phosphate buffer of 10 mM concentration (pH 7). The gate voltage shift suggests increased sensitivity of the poly-Si NWFET. 

Another selectivity sensor challenge is closely related to non-specific binding (NSB), a common issue faced by nanomaterial-based sensors, because a small degree of NSB affects the sensor sensitivity substantially. This can be mitigated to a certain extent by the use of PEG-containing polymers as the coating layer on the FET channel surface. The sensing receptors can be affixed on the PEG-coated surface covalently. The PEG-coating provides a high hydrophilic surface and is charge-neutral, thus banishing the hydrophobic interaction of unwanted ions on the FET channel surface. However, this may change the FET channel surface properties [71].

The rapid growth of microfluidic technologies has made the lab-on-a-chip (LoC) FET sensor possible. In general, the system requires fluidic handling to transport the solution of interest to the reaction zone and subsequently makes contact with the FET sensor receptors or probes to selectively capture the target. A transduction mechanism is required to convert the FET sensor responses to electrical signals for the end user to view the results. However, to get an accurate analysis, sample preparation and pre-treatment are necessary for this system. Sample preparation is essential to remove contaminant ions in the analyte of interest and to extract target analytes. A major challenge in microfluidic systems is the complexity of the sample preparation. Too large particles may clog up the micron-sized microfluidic channel. In real-life applications, one size microfluidic channel does not fit all heavy metal ions. Non-specific absorption on the microfluidic wall is another concern that needs to be addressed, because it may result in non-specific signals, changes in flow behavior, and channel clogging [72]. Beyond that, the integration of the microfluidic system and the FET sensor must be seamless to justify the claims of high sensitivity and rapid response times, as this includes the sample preparation period. In regard to sensor signal reading, choosing a strategy for signal amplification is quite a hurdle, as the presence of the target is mostly at trace levels. The amplification strategies have been highlighted in the literature [73,74]. Although the amplification strategy does increase sensor sensitivity, nevertheless, this comes at the expense of an increase in analysis time and complexity. Moreover, an amplification strategy with complex procedures tends to increase the chance of errors and reduce reliability.

Another issue is real-time measurement without cleaning the sensor surface or changing the sensor. Besides the specific target binding, NSB from analyte components tends to adhere to the sensor surface. Both specific target binding and NSB give rise to biofouling, which forms an impermeable layer that inhibits the sensor surface, resulting in false readings and impeding the sensor’s reusability. Biofouling affects sensor performance in terms of sensitivity, stability, reproducibility and reliability [75]. In any case, the FET sensor requires anti-fouling strategies or a self-cleaning surface to avoid surface effects for real-time measurement. A number of publications have proposed anti-fouling strategies to alleviate the biofouling problem on the FET sensor channel surface. For example, Yang et al. used covalent organic frameworks (COFs) to encapsulate the FET sensor channel to prevent it from being fouled [76]. COFs are crystalline mesoporous polymers [77] that allow modification and functionalization on their large specific surface area. Yang et al. reported that the FET sensor performance with anti-fouling COFs was comparable to that of a FET sensor without anti-fouling property. This anti-fouling approach is convenient for FET sensor construction. Another anti-fouling strategy is to separate the sensing surface that accommodates the target analytes from the measuring surface. However, this strategy is only applicable for FET sensors with side gate [78], floating gate [79,80] and dual-gate [81,82]. As an alternative to typical FET sensor construction, a semipermeable lipid membrane coating is used on the FET channel to separate the sensing surface from the target analytes and only allow the target of interest to reach the sensing area [83]. Deployment of the FET device as a monitoring tool for HMIs has to consider self-cleaning sensing surface for long-term use and reusability without compromising its performance. An advantage of the self-cleaning sensor is that it helps to avoid tedious treatment after being used. It is feasible by modifying the sensor transducer with a superhydrophobic and conductive nanocomposite. The nanocomposite superhydrophobicity property helps to mitigate absorption from molecules and oxidation in air, whereas its high conductivity property is very useful for enhancing sensor signals. Zhu et al. fabricated a self-cleaning sensor by implementing a superhydrophobic and conductive nanocomposite of polydimethylsiloxane (PDMS) and multiwall carbon nanotubes (MWCNT) on the sensor electrode [84]. They reported that the sensor electrode integrated with PDMS@MWCNT can be shelved for more than one month with no signal degradation and significant passivation. The sensor is reusable by simple washing. Besides conductive material, PDMS can be grafted with zwitterionic copolymer as a self-cleaning surface material. De Vera et al. grafted PDMS material onto poly (glycidyl methacrylate-co-sulfobetaine methacrylate-co-2-(dimethylamino) ethylmethacrylate) polymer, poly (GMA-co-SBMA-co-DMAEMA) to develop pH dependent self-cleaning surface [85]. Their findings confirmed the reversible attachment and detachment of microorganisms, which paved the way for reusable biosensors. However, the pH target analytes must be reconsidered as this material is pH dependent; therefore, the ionic strength of the analytes may affect the sensor response.

## 4. Silicon-Based FET

Silicon (Si) semiconductor has drawn great attention as sensor material due to its abundance availability and surface modification flexibility [86]. In addition, silicon can be manipulated into different nanostructures, such as nanowires, nanorods and nanoporous, which offer large surface areas to produce high sensitivity sensors [87]. Moreover, mature silicon technologies enable seamless integration with complementary metal-oxide semiconductor (CMOS) circuits for direct sensing systems [88]. Si materials have shown promise towards the evolution of FET biosensors since the seminal work by Bergveld in the 1970s [15]. To date, considerable efforts have been made to develop a reliable and high sensitivity silicon-based FET sensor for various ions’ detection, including heavy metal ions. 

**Figure 4:**
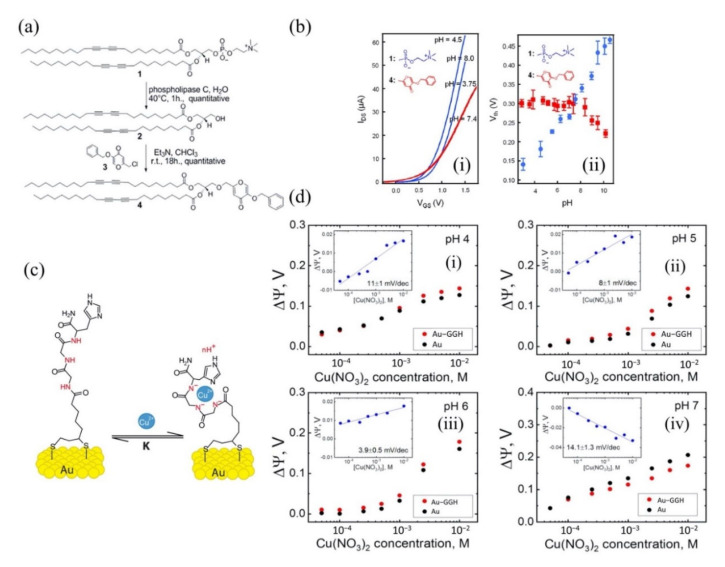
pH insensitive sensing lipid for detection Fe^3+^ ions (**a**) A schematic of the hydroxyl γ-pyrone derivatives lipid engineered by Nguyen et al. group. (**b**-**i**) and (**b**-**ii**) comparison between commercial DCPC lipid (blue) and Nguyen’s modified lipid (red) behavior in pH solutions. Reprinted from [92], Copyright 2013, with permission from Elsevier. SiNR-FET sensor functionalized with Gly–Gly–His (GGH) for detection Cu^2+^ (**c**) GGH tethered on gold structure and (**d-i**–**iv**) sensor response at pH 4,5,6 and 7, respectively. Reproduced with permission from [104]. Copyright 2019 MDPI.

Nguyen et al. and co-workers investigated the potential of hydroxypyridinone derivative modified lipid as an active sensing layer on silicon FET for ferric ions (Fe^3+^) detection [89]. In this work, Nguyen used pyridinone-phospholipase (an enzyme) to modify an ultrathin (~3 nm) organic lipid to create a receptor compound that selectively binds with Fe^3+^. Subsequently, the modified lipid was absorbed onto the FET channel surface (Si-H) using the vesicle fusion technique, which is widely employed to create lipid layers [90]. Upon Fe^3+^ detection, the FET gate-source voltage shifted up to 200 mV, suggesting good sensitivity of the sensor. The most important finding was that the pyridinone-embedded FET sensor exhibited specific detection of Fe^3+^ in a wide detection range of 5 pM to 50 µM. The remarkable detection results are attributed to the high binding capability of hydroxypyridinone with ferric ions (Fe^3+^), which is in good agreement with a published article by Chaves et al. [91]. Further, the research group investigated γ-pyrone derivative engineered lipid as a pH-independent sensing layer on silicon FET for Fe^3+^ detection [92]. A commercial lipid was cleaved with an enzyme before being tethered to γ-pyrone for ferric ion detection. The thickness of the lipid layer was reduced to 2.7 nm to improve the sensor sensitivity. The diagram of the modified γ-pyrone derivative lipid layer is illustrated as in Figure 4a. The results of this study showed that the γ-pyrone monolayer lipid was pH-insensitive. The electrical property of the modified lipid layer was examined in different pH solutions. A commercial DCPC lipid was used in this experiment as a comparison to Nguyen’s γ-pyrone modified lipid. Figure 4b(i,ii) compare the lipid behavior in different pH solutions (pH 2–10). The DCPC lipid (blue) shows the current and V_TH_ change across the pH, while Nguyen’s γ-pyrone modified lipid (red) exhibits a stable current and no V_TH_ shift, especially in the pH range of 2 to 7. In their sensing studies, the γ-pyrone modified lipid FET sensor showed a relative response to Fe^3+^. This finding broadly supports the work of other studies in this area, linking the high affinity of γ-pyrone towards iron ions [93,94]. Their experiment data conclusively shows that the sensor response was not under pH influence and the modified γ-pyrone lipid layer is suitable to be used as a FET sensing element in medical diagnostic applications such as blood, sweat and urea, in which pH variations tend to occur, especially when patients are treated with drugs. This sensing strategy can discriminate Fe^3+^ from interfering ions in a concentrated solution as high as 50 GM and as low as 50 fM (detection limit). In another report, a modified lipid with di-2-picolylamine (DPA) was embedded in an FET channel for Cu^2+^ detection in a solution [95]. This is the first report showing a dual-gate FET sensor with engineered lipid monolayer for HMI detection. The lipid used in this assay was engineered to prevent ion trapping and provided sensor stability. The assay exhibited remarkable sensing potential with sensitivity of 98 ± 3 mV/decade, which exceeds the Nernst limit of 29.5 mV/decade predicted for divalent ion by a factor of three. The authors claimed that this super-Nernstian performance was attributed to dual-gate FET geometry, as the performance of their previous single-gated FET sensor merely exhibited Nernstian behavior [92]. Furthermore, this lipid FET sensor demonstrated exceptionally low limit detection at the femtomolar level (10 fM) and had sufficient specificity towards Cu^2+^.

A few nanowire silicon-based FET sensors for heavy metal detection have also been published in the literature. The majority of recent work in this field stems from the seminal work of Cui et al., who first explored the potential of SiNWs as a highly sensitive biosensor [96]. Cui and co-workers primarily demonstrated vapor-liquid-solid (VLS) grown SiNWs FET for ion detection, and later they carried out the experiment for the detection of streptavidin binding on the biotin-functionalized SiNWs surface. Their research on SiNWs as biomolecular sensors was extended to the detection of calcium ions (Ca^2+^). The SiNWs surface was immobilized with calmodulin as a receptor to recognize the Ca^2+^ ions. These studies have demonstrated the high potential of SiNWs as a material for biosensors. In recent years, there has been an increasing interest in SiNWs-FET for HMIs detection. Jin et al. demonstrated a free-standing SiNWs-based FET sensor that can detect mercury ion (Hg^2+^) as low as 4.985 mM [97] and has a wide detection window, in a linear range (R^2^ = 0.9838) between 4.985 mM and 24.926 nM. The sensors were fabricated in an array using the top-down method, which demonstrated the ability to exhibit high selectivity toward Hg^2+^ ions in real-time detection. The SiNWs electrode surface was functionalized with (3-Mercaptopropyl) trimethoxysilane (MPTMS), an organosilane with a thiol group as receptor to recognize Hg^2+^ ions. More interesting is that the type of SiNWs used in this investigation were inverted-triangle-shaped. With a highly controllable microfabrication process, the diameter size of the free-standing SiNWs was determined around 100 nm. This SiNWs geometry provided advantages in using all three facets as sensing areas. Therefore, this sensor configuration provided more thiol probes to bind with Hg^2+^ ions. Accordingly, the performance of the sensor was enhanced. Even though the sensor shows excellent sensitivity and selectivity, the reproducibility and stability data of the sensor were lacking. To address the stability issue, Huang et al. [98] from the same research group produced a better and more stable SiNWs FET-based sensor for the detection of Hg^2+^. The sensing strategy for this sensor was a little bit different than the previous sensor. With the aim of providing more thiols as sensing probes, the new sensor utilized gold nanoparticles (GNP), which were functionalized on the SiNWs surface with the aid of 3-Aminopropyltriethoxysilane (APTES). Subsequently, the thiol groups were immobilized on top of GNPs as receptors to bind with Hg^2+^ ions. The use of GNPs has increased the surface area and the number of thiol probes to amplify the response signal. The resulting sensor exhibited high selectivity and ultra-sensitivity of Hg^2+^ ions with a LOD of 0.06 pM and a wide range of detection of 1 ng/L–10 μg/L^−1^. Additionally, this sensor also demonstrated very fast response in unknown samples under 1 min. The use of DNAzyme on the SiNW-FET channel as a receptor for HMI recognition has also been reported with promising results. A simple approach for selective detection of silver ions (Ag^2+^) in aqueous on the basis of C-Ag^2+^-C coordination chemistry was proposed by Chen et al. [99]. The silver specific oligonucleotide (SSO), which richly contains cytosine mismatched base pairs (C-C), was grafted onto the SiNW FET device. When Ag^2+^ is introduced into the solution, the mechanism is primarily based on the formation of stable cytosine-Ag^2+^-cytosine, which converts a single-stranded SSO into a double helix structure. The helix structure accordingly increased the negative charges on the SiNWs surface, resulting in the conductance increased in gate oxide. With this sensing strategy, authors reported the detection of Ag^2+^ could be up to 100 μM, while the LOD of this sensor was 3 nM. Moreover, the DNAzyme/SiNWs FET sensor demonstrated a linear response with increasing Ag^2+^ concentration.

**Figure 5:**
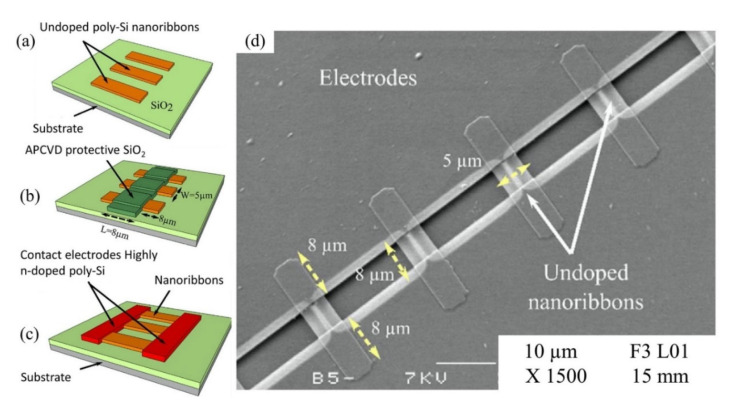
Construction of resistor sensor for Pb^2+^ (**a**) Deposition of poly-Si nanoribbons electrode on silicon wafer substrate (**b**) Deposition of insulating layer of SiO_2_ by atmospheric pressure chemical vapor deposition (APCVD) (**c**) Deposition of contact electrodes on poly-Si nanoribbons and removal of the SiO_2_ protective layer and (**d**) SEM image (top view) of the the resistor sensor. Reprinted from [105], Copyright 2018, with permission from Elsevier.

Other than SiNWs, silicon nanoribbons (SiNRs) are also receiving a significant amount of attention for biosensor fabrication [100,101,102]. It is a great alternative to SiNWs as a sensor transducer material because it offers compatibility with the top down CMOS process [103]. A recent example of an electrical assay to detect HMI with SiNRs was demonstrated by Synhaivska et al. The research group has designed a SiNRs ISFET functionalized with glysine–glysine–histidine (Gly–Gly–His, GGH), as illustrated in Figure 4c, for the detection of copper ions (Cu^2+^) [104]. The detection of the Cu^2+^ using GGH ligands is based on the conformational change of the ligands when the secondary amides are deprotonated in the presence of copper ions, leading to increases of negative ions near the SiNR surface. Figure 4d(i–iv) shows the response of the GGH functionalized SiNR FET sensor in high concentration Cu(NO_3_)_2_ solutions (0.05–10 mM) at pH 4, 5, 6, and 7. The sensitivity of the sensor at pH 4, 5, 6, and 7 was 11, 8, 3.9, and 14.1 mV/dec, respectively. These findings suggest that the sensor performance was pH dependent, and these observed results could be attributed to the protonation and deprotonation degrees of secondary amides at different pH. Interestingly, the GGH/SiNR-FET sensor gave a negative differential response in a neutral solution (pH 7), as seen from Figure 4d(iv). A possible explanation for this result was that in pH 7 solution, the Cu^2+^ ions dominated the interaction on the FET channel surface over hydroxyl (OH^-^) or hydrogen (H^+^) ions. The FET sensor demonstrated linear responses in varying Cu^2+^ concentrations (from 0.1 fM to 0.1 μM) at pH 7 solutions and reached LOD at 10 fM, which is exceptionally lower than any other HMI FET sensor that has been mentioned in this review. However, the main drawback of this sensing approach is that the deprotonation of the secondary amides in GGH by the pH media can limit the sensor application. Also, a poly-silicon nanoribbon resistor sensor grafted with aryl diazonium salts for sensing Pb^2+^ has been proposed [105]. Similar to a FET sensor, the resistor or chemiresistor type sensor changes its electrical resistance corresponds to potential changes on its channel surface, except that it does not involve field-effect to control the conductivity in the channel region. Construction of the poly-Si nanoribbon resistor sensor for Pb^2+^ sensing is shown in Figure 5a–c. The SEM image in Figure 5d shows the top view of the resistor sensor with a length and width of 8 µm and 5 µm, respectively. The sensing mechanism of this sensor relies on the positively charged Pb^2+^ ions that are attracted to negatively charged carboxylate groups (-COOH) on the SiNR surface, resulting in modulation of the quantity of charge flowing through the resistor. The surface functionalization of the poly-silicon nanoribbon FET is relatively easy. A simple dip in aqueous solutions that contain the recognition element for a certain period can give homogenous functionalization coverage on the silicon surface, in this case, aryl diazonium salts. The resistor sensor successfully detected the Pb^2+^ in a variety of concentrations, from 10 µM to 1 µM. Both of the poly-SiNR sensor devices reviewed in this paper are functionalization of the surface employed by the spontaneous grafting technique. Although the grafting method for SiNR surface functionalization is straightforward, the long exposure time of the SiNR-FET in the receptor contained solution could lead to over functionalization, which may produce a screening effect [106].

Silicon-alloy materials such as silicon carbide (SiC), silicon nitride (Si_3_N_4_), and silicon germanium (SiGe) have also been described for FET sensor development to detect biomolecules, ions, and DNA [107,108,109]. Although considerable advancements have been achieved in ion detection, the silicon-alloy-based FET sensor is scarcely explored for HMIs. From our observation, the research in this field has tended to focus on silicon nanostructures rather than silicon-alloy as a sensor material. This focus is because the dimensional similarity of the nanostructures and the subject to sense offers seamless integration of nanoelectronics and life sciences, which enables high sensitivity and revolutionizes the sensing and detection area.

## 5. Carbon-Based FETs

### 5.1. Carbon Nanotubes (CNTs) FET

Since its discovery in 1991 by Ijima [110], CNT material has attracted significant attention in scientific research. At the time, CNTs immediately became one of the promising materials for biosensor transducers owing to their physical and electrical properties. CNTs and graphene share many excellent properties, as CNTs are made of single or multiple layers of graphene rolled around seamlessly with the advantage of having an endless, perfect hexagonal structure. CNTs are a popular material with a large surface-to-volume ratio, which makes them a good semiconductor for high sensitivity sensors. One molecule of absorption on the sidewall of CNTs promotes a change in the local electrostatic environment, consequently changing its conductance [111]. Furthermore, its unique electronic transport properties have high potential for sensor device miniaturization. Its atomically thin structure gives it an advantage as an ideal electrostatic control over the channel, which is important for a miniature device sensor [112]. Additionally, on the account of its biocompatibility properties and size compatibility with a single molecule make CNTs an ideal platform for biosensing materials [113]. A large volume of literature has reported the application of carbon nanotube FETs for bio- and chemical sensors [114,115,116]. Over time, the CNT-based FET sensor has been constantly fabricated and its potential has been explored for different target detection.

RNA-cleaving DNAzyme is commonly employed for sensitive and selective HMI detection in biosensors or chemical sensors. DNAzyme possesses unique chemical properties that selectively bind to specific metal ions with multiple turnovers, making it an ideal recognition element [117]. DNAzyme in general consists of an enzyme strand and a substrate strand. The latter accommodates a single ribonucleobase (RNA) linkage functioning as a cleavage site. In the absence of targeted metal ions (cofactor), these two strands are hybridized to form a double helix in buffer solution. While in the presence of the target metal ions, DNAzyme cleaves the substrate strand, releasing a shorter fragment from the duplex. The change in DNAzyme structure can be used to induce the carrier accumulation numbers in nanomaterials. Thereby, DNAzyme is very convenient to be used as an FET sensing probe. DNAzyme being used in other sensing platforms has recently been reviewed elsewhere [118,119].

**Figure 6:**
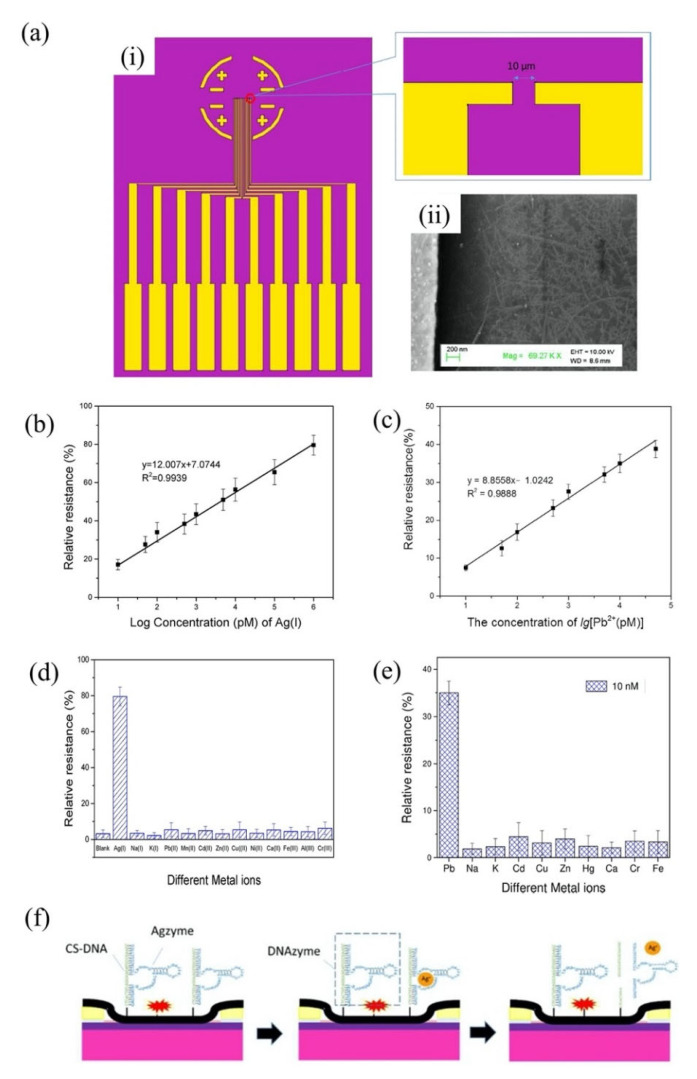
DNAzyme/SWCNTs/FET device proposed by Wang et al. (**a**-**i**) There are five FET devices on a chip. The size of each channel width was 10µm (a-ii) SEM image of SWCNTs FET. (**b**) The linearity response of the Agzyme/SWCNTs/FET with increasing Ag^+^ concentration. Reproduced with permission from [120]. Copyright 2018 MDPI. (**c**) The linearity response of the Pbzyme/SWCNTs/FET with increasing Pb^2+^ concentration. Reprinted with permission from reference [121]. Copyright 2019 John Wiley and Sons. (**d**) Selectivity performance of Agzyme/SWCNTs/FET in interference ions solutions. Reproduced with permission from [120]. Copyright 2018 MDPI. (**e**) Selectivity performance of Pbzyme/SWCNTs/FET in interference ions solutions. Reprinted with permission from reference [121]. Copyright 2019 John Wiley and Sons. (**f**) DNAzyme cleaves its substrate strand at RNA site in the presence of HMIs. Reproduced with permission from [120]. Copyright 2018 MDPI.

The group of Wang et al. has made a tremendous contribution to the detection of HMIs based on the single-walled carbon nanotubes (SWCNT)-FET sensor platform. Most of his HMI detection work exploited metal-dependent DNAzyme and aptamer as sensing elements on SWCNT-FET sensors. In 2018, the research group demonstrated a CNT-based FET functionalized with silver specific RNA-cleaving DNAzyme for the detection of silver ions (Ag^+^) [120]. The FET structure and SEM image of the sensor are shown in Figure 6a(i,ii), respectively. In the presence of Ag^+^, the substrate strand was fragmented and released from the RNA-base, leading to a change in DNAzyme’s structural and, subsequently, the change the electrical conductivity of the CNT-FET. This sensor exhibited high sensitivity and selectivity towards Ag^+^ at a linear response range of 10^1^ pM to 10^6^ pM (Figure 6b). The Agzyme/SWNTs-FET sensor’s selectivity was determined by testing the sensor response in various ions found in water environments, including Na^+^, K^+^, Pb^2+^, Mn^2+^, Cd^2+^, Zn^2+,^ Cu^2+,^ Ni^2+^, Ca^2+^, Fe^3+^, Al^3+^ and Cr^3+^. The result indicated that the Agzyme/SWNTs-FET sensor exhibited the highest relative resistance change in Ag^+^ solution, suggesting that it has excellent selectivity towards Ag^+^. The Agzyme/SWNTs-FET demonstrated resistance to change in the Pb^2+^, Cd^2+^ and Cu^2+^ solutions, but the values were less than 5%, as shown in Figure 6d. The limit of detection of 5 pM was obtained. Further, the Agzyme/SWNTs-FET was investigated in a real river water sample. Table 2 shows the results of Ag^+^ analysis in the river water measured by Agzyme/SWNTs-FET. An atomic absorption/emission spectroscopy (AAS) system was used as a gauge in this experiment. The findings showed that the sensitivity of the Agzyme/SWNTs-FET sensor was comparable with the AAS readings, though it must be pre-treated prior to sensing to remove the undissolved substances in the river water sample. Besides, the Agzyme/SWNTs-FET exhibited a significantly high recovery response that ranged from 92.45% to 105.12% which implied the proposed sensor can quantify the Ag^+^ ions in real river water with acceptable accuracy. Wang and colleagues also demonstrated new strategies for electrical detection of Pb^2+^ concerning the potential of GR-5 DNAzyme (Pbzyme) [121] which could cleave the substrate strand at the RNA site in the presence of Pb^2+^ [122,123]. The sensor structure was similar to the previously mentioned sensor for detection of Ag^+^ (Figure 6a). An ultrasensitive SWCNT-FET sensor functionalized with GR-5 DNAzyme reported a good linear range detection from 10 pM to 50 pM with a LOD of 7.4 pM. Figure 6c explains the linearity of the sensor response as Pb^2+^ concentrations in solutions increase. The Pbzyme/SWCNT-FET sensor exhibited excellent Pb^2+^ discrimination in the electrolyte, due to its higher affinity for Pb^2+^ over the other competitor ions, as seen from the bar graph in Figure 6e. Although we noted that the relative resistance of Pbzyme/SWCNT-FET also slightly changed when tested in Cd^2+^, Zn^2+^, Cr^3+^ and Fe^3+^, solutions, the sensor resistance change with these interference ions was much less than the resistance change in Pb^2+^ solution. The sensor was then used to quantify Pb^2+^ in woodland soil and paint samples and gave comparable readings to the atomic emission spectrometry (AES) system. Table 3 compares the Pb^2+^ analysis in woodland soil and paint results obtained by the Pbzyme/SWCNT-FET and AES. The readings by Pbzyme/SWCNT-FET show the measurement error is relatively lower than 10% compared to the AES measurement system across the eight samples, which is sufficiently acceptable. This data concludes that the analysis by Pbzyme/SWCNT-FET is accurate and reproducible. The DNAzyme structural change and RNA cleaving mechanism with the presence of HMIs in Wang’s experiments can be described as in Figure 6f. The DNAzyme structural change on the FET channel altered the CNT surface potential, subsequently changing the density of carrier concentration of CNT material.

Noting that not all the metal ions are sensitive and selectively bind with DNAzyme, detection of other heavy metal ions that do not have specific DNAzyme can remain challenging. For example, detection of Cu^2+^ with a DNAzyme strategy is difficult, especially in a complex matrix because of interference from Hg^2+^ ions. Due to extremely high thiophilicity, the Hg^2+^ secludes the Cu^2+^ forming a stabilized complex with DNAyzme. Therefore, the sensing of Cu^2+^ becomes difficult with the presence of Hg^2+^ at the same time. To address this issue, a SWCNT-FET based biosensor array was proposed for the determination of Cu^2+^ and Hg^2+^ using Gaussian process regression (GPR) [124]. The SWCNT surface was immobilized with DNAzyme and its complementary DNA, denoted as PSCu10 and complementary DNA embedded phosphorothioate RNA (CS-DNA), respectively, for recognition of Cu^2+^. The principle of action of this sensor is similar to any other RNA-cleaving concept that is described earlier. The formation of Cu^2+^ with the PSCu10 DNAzyme (enzyme strand) facilitates the cleavage of CS-DNA (substrate strand) at the RNA site. In this work, Gaussian process regression [125] was proposed to build a prediction model to estimate the Cu^2+^ concentration. Both Hgzyme (mercury specific DNAzyme) and PSCu10 DNAzyme were immobilized on the SWCNT-FET sensor electrode with their respective substrate strands. To investigate the sensor response towards targeted Cu^2+^ and Hg^2+^, the biosensor array was immersed in solutions with different Cu^2+^ and Hg^2+^ concentrations ranging from 0.01 to 10,000 nM. The findings show that the percentage of relative resistance was constantly increased with the ion concentration in the case of Cu^2+^. In contrast to the response of Hg^2+^, the relative resistance shows an increasing trend starting from 5 nM to 10,000 nM. This sensor provided LOD Cu^2+^ and Hg^2+^ with 6.7 pM and 3.43 nM, respectively. The accuracy of the prediction of Cu^2+^ concentration was indicated by a correlation coefficient (R_0_) of 0.985 and the root mean square error between the actual Cu^2+^ ion concentration and the forecasted Cu^2+^ concentration, which was 0.038. Recently, an interesting SWCNT-FET sensor was developed using a similar sensing approach for detection and monitoring of Cd^2+^ in feed [126]. Similar to Cu^2+^, the determination of Cd^2+^ ions’ DNAzyme strategy is rather difficult because it lacks specific DNAzymes, and Cd^2+^ recognition can be hindered by interference from multiple other metal ions, such as Hg^2+^ and Pb^2+^. The SWCNT-FET sensors were fabricated in an array and for each sensor’s channel surface was immobilized with three different types of DNAzymes (Cdzyme, Hgzyme and Pbzyme) with their respective substrate strand. The percentage of resistance data was collected before continuing with Gaussian process regression. This sensor reached its LOD at 3.4 × 10^−2^ nM. Although the study was successfully demonstrated, SWCNT-FET functionalized with non-specific DNAzyme combining GPR prediction could be used as a sensor to identify a specific metal ion in a complex matrix solution, but it is still necessary to develop a more general method to realize various sensing targets.

**Figure 7:**
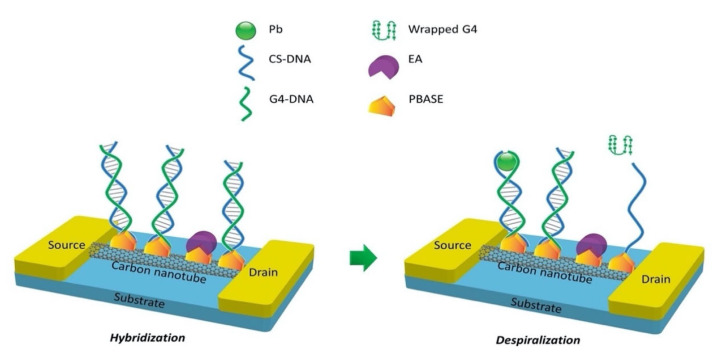
Hybridization and despiralization of G4-DNA and CS-DNA induced conductivity of CNT-FET sensor.

Albeit DNAzyme has high sensitivity and selectivity towards a specific HMI, its structure is destroyed in the sensing process (substrate strand is cleaved at the RNA site). Therefore, the reusability of the DNAzyme/FET sensor over a long period of time is impossible. The FET functionalized aptamer was investigated as an alternative probe to detect HMIs. Compared to DNAzyme, aptamer (a single-stranded DNA) is a better choice for sensor recognition elements as the aptamer only changes its structure in the presence of the target. An FET sensor functionalized with G-quadraplex aptamer (G4-DNA) and complementary CS-DNA has been developed for the determination of Pb^2+^ ions and shows promising for reusable FET sensors [127]. As remarked in previous literature, G4-DNA was identified as a functional DNA molecule that has a specific binding affinity for Pb^2+^ [128,129]. The main mechanism of the sensor relied on the efficiency of the Pb^2+^ ions to induce conformational changes in G4-DNA. In the Pb^2+^ absence, the G4-DNA and CS-DNA were hybridized to form double-stranded DNA (duplex DNA). While in the presence of Pb^2+^ ions, the duplex DNA was despiralized by the Pb^2+^ to establish G4/Pb^2+^ as a stable complex. The sensing mechanism of the aptamer was illustrated as in Figure 7. The structural change of the aptamer (hybridization and despiralization) was used as signaling for detection of Pb^2+^ on the FET channel surface. The aptamer conformational change had induced the conductivity of the SWCNT, resulting in a resistivity decrease. This sensor provided a wide linear detection range from 1 ng/L to 100,000 ng/L (R^2^ = 0.9902) and a low LOD of 0.39 ng/L. This sensing approach shows stability, good reproducibility, high sensitivity and selectivity towards Pb^2+^ ions. 

The development of antibodies-antigen based sensors or immunoassays for the detection of heavy metal ions has grown rapidly since the discovery of antibodies against metal chelates in 1985 [130]. To date, a total of 66 antibodies against metal ions have been reported in the published literature [131]. An SWCNT-FET immunoassay functionalized with transferrin antibody for detection of iron (III) in wine has been reported [132]. The transferrin antibodies were non-covalently immobilized and directly absorbed into the SWCNT-FET channel area. The sensing mechanism of this sensor was based on the chelation interaction between Fe^3+^ and chelating ligands of siderophores to form ferric-siderophores complexes. LOD of <0.05 ng mL^−1^ was reported with a linear range from 0.05 to 2 ng mL^−1^. The sensor was evaluated in a wine sample and yielded high accuracy in Fe^3+^ detection without pre-treatment.

### 5.2. Graphene FET

Graphene is a zero bandgap semiconductor [133] that emerged as a powerful platform for biosensors due to its unique properties, such as biocompatibility [134] and remarkable high signal-to-noise ratio (SNR) [135], and versatility for surface functionalization. It also has significant potential for mass production and is possible for miniaturization. Graphene also offers direct detection on its functionalized surface, which eliminates the need for additional membrane layers required for sensing purposes. Additionally, graphene is ideal for sensing platforms because of its high surface area, contributing to the sensor’s high sensitivity and selectivity [136].

Thiol group (-SH) are easily chelated with Cd, Zn, Cu, Hg, Au, Ag, Bi and Co because of their high affinity with HMIs. Zhang and co-workers [137] have developed a simple electrical detection approach for Hg^2+^ determination using graphene FET (GFET), functionalized with 1-Octadecanethiol. Zhang’s work was inspired by previous studies of the absorption of long substituted alkyl chains on highly ordered pyrolytic graphite (HOPG) [138,139,140]. The sensing mechanism of their sensor lies in the chelation interaction between Hg^2+^ and -SH groups. Consequently, this induces the graphene carrier, resulting in the V_DIRAC_ shift, confirming the bonding of Hg^2+^ with thiols on the graphene surface. They reported that the GFET sensor had high reproducibility as the other seven devices showed an average V_DIRAC_ shift of (6.2 ± 2.0) V. This sensing approach successfully detected Hg^2+^ at 4.985 nM. As a comparative study to Zhang’s earlier work, Nasima et al. investigated the effect of two different alkanethiol surface functionalizations on the determination of Hg^2+^ and Pb^2+^ ions with a GFET sensor [141]. The research group functionalized the channel surface of the sensors with 1-Octadecanethiol and 1-Dodecanethiol, separately. On the 1-Octadecanethiol surface, the authors reported that their GFET sensor responded towards Hg^2+^ and Pb^2+^ ions and the detection of both ions were comparable with Zhang’s data (Hg^2+^ = 49.85 µM and Pb^2+^ = 48.263). It was observed that the average V_DIRAC_ shift for Pb^2+^ was larger than that of Hg^2+^, possibly due to the stronger affinity binding between thiols-Pb^2+^ than thiols-Hg^2+^ [142]. However, the 1-Dodecanethiol functionalized GFET exhibited no shifts in V_DIRAC_, complicating its application as an HMI probe. Even though both studies successfully demonstrated reproducibility, reliability, and consistency of detection, the papers lack the study of selectivity sensor performances.

The metal-DNA complex formation could be useful as a nanomaterial biosensor signaling for target detection. With one-end of single-strand DNA (ssDNA) confines directly on the graphene surface or any other linker (i.e., gold nanoparticles (AuNPs)), while the other end binds with HMIs based on affinity interaction. In the presence of HMIs, the ssDNA will go through a conformational and structural change. For example, an electrical detection of Pb^2+^ ions using guanine-rich DNA (G-rich DNA) has been proposed [143] in which the G-rich DNA was tethered to AuNPs before linking to the graphene surface. Owing to AuNPs’ high surface-to-volume ratio, it is an exceptional molecular scaffold or adaptor for nanosensors, which could provide additional sites for intended target probes, which could then improve the sensitivity of the detection [144]. In the presence of Pb^2+^, the G-rich DNA would transform into a G-quadraplex structure by wrapping itself around the ion. Benefiting from the high affinity of the G-rich DNA toward Pb^2+^ ions [145], this sensor exhibits excellent sensing performance with detection as low as 20 nM, which is significantly lower than the guideline set by the World Health Organization (WHO) for the maximum level of Pb^2+^ ions in drinking water (50 nM limit). Li et al. carried out similar studies on a labelled-free Pb^2+^ sensor with GFET functionalized with G-rich DNA [146]. In this work, G-rich DNA was affixed directly on the graphene surface. The ssDNA bears negative charges [147], thereby leading to the accumulation of holes in the GFET channel. When interacting with Pb^2+^ in an electrolyte, the DNA will collapse to transform into a G-quadraplex structure, bringing more charged nucleotides to induce holes in the graphene conduction band. With a LOD level down to 163.7 g/L, this experiment has confirmed that Pb^2+^ ions strongly and selectively bound to the G-rich strand.

**Figure 8:**
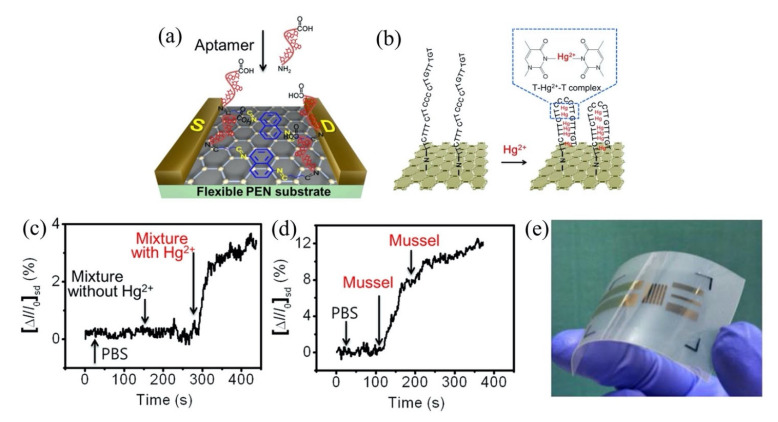
Graphene FET sensor for detection Hg^2+^ in mussels. (**a**) Aptamer (30-amine-TTC TTT CTT CCC CTT GTT TGT-C10 carboxylic acid-50) was functionalized on graphene FET channel surface. (**b**) Interaction of Hg^2+^ with thymine base pair. (**c**) Real-time FET sensor response in a solution containing Hg^2+^ ions. (**d**) Real-time FET sensor response in a real sample mussels’ solution. (**e**) Flexible, transparent and lightweight graphene FET sensor device. Reprinted (adapted) with permission from [149]. Copyright 2013 American Chemical Society.

Moreover, the use of aptamer to detect heavy metal ions has gained more interest in recent years due to its reliability and chemical simplicity. Tu et al. [148] recently described an approach to yield a consistent response in GFET and its further use for electrical detection of heavy metal ions, particularly by the fabrication of an array of GFETs with ssDNA aptamer modification on the channel area for detection of Hg^2+^. The GFET array was comprised of 36 common-source FETs, resulting in a larger current response and a consistent sensing result. The sensor exhibited a wide detection range from 100 pM to 100 nM and a low detection limit of 40 pM. Remarkably, this sensor’s response time was less than 1 s and had achieved rapid detection of HMIs. Likewise, An et al. used (30-amine-TTC TTT CTT CCC CTT GTT TGT-C10 carboxylic acid-50) aptamer on a flexible graphene surface aided by 1,5-diaminonaphthalene (DAN) and glutaral-dehyde (GA) to determine Hg^2+^ in a mussel [149], an organism that contains a variety of heavy metals including Hg^2+^ [150]. The aptamer was immobilized on the graphene FET channel surface through 1,5-diaminonaphthalene (denoted as blue hexagon) as a linker, as shown in Figure 8a. The interaction of Hg^2+^ with thymine base pair to form a thermally stabilized duplex structure of T-Hg^2+^-T has induced the conductivity of the FET sensor. Figure 8b depicts the formation of the T-Hg^2+^-T coordination on the graphene. Adding Hg^2+^ to the graphene FET sensing area (channel) led to the carrier of holes near the graphene surface increasing, which subsequently resulted in the increase of FET drain current. This sensor obtained a LOD of 10 pM and exhibited an exceptionally low response time below 1 s. As a confirmatory analysis, the sensor was tested in mussels’ samples that contained a 0.3749 mM concentration of Hg^2+^ from nature and demonstrated a fast response of Hg^2+^ mercury discrimination in the complex matrices. Figure 8c,d displays the real-time detection of Hg^2+^ in a mixture of solutions and in mussel solutions, respectively. Both experimental results show the current signal change when the FET sensor was exposed to the solutions containing Hg^2+^. The aptamer/graphene-FET sensor proposed by An et al. was flexible and lightweight, offering portability, and is suitable for on-site application as shown in Figure 8e. The group of Li et al. investigated the potential of reduced carboxyl graphene oxide (rGO-COOH) as a screen-printed graphene FET sensor. They further modified the FET active channel with a lead-specific aptamer (LSA) to detect Pb^2+^ [151]. It is noteworthy that the LSA aptamer was attached directly onto the rGO-COOH surface without any linker. The carbon rings of graphene and the ring atoms of nucleobases LSA aptamer were bound through hydrophobic and π-stacking interactions [152,153]. The detection of the Pb^2+^ can be observed by the increase in drain current caused by the cleavage effect of the substrate strand. Specificity for Pb^2+^ was achieved in real sample drinking water and a detection limit of 4.826 pM was obtained.

The use of DNAzyme probes for heavy metal detection (mainly Pb^2+^ and Hg^2+^) is established based on two phenomena: (a) RNA-cleaving in the presence of the metal ions and (b) affinity for metal ions (cofactor). Based on this principle, Wen et al. developed a novel DNAzyme/graphene FET sensor to identify Pb^2+^ ions [154]. The lead dependent DNAzymes were anchored on the graphene site through AuNPs. The Pb^2+^-dependent DNAzyme is comprised of an enzyme strand (17E) and a thiolated substrate strand (17S). The enzyme strand cleaves the substrate strand when the Pb^2+^ is present. Immediately, the enzymatic strand and the shorter cleaved substrate strand fragment leave the DNAzyme structure [155,156]. These DNAzyme conformational changes certainly altered the electronic properties of the graphene, similar to what has been discussed in the CNT section (Figure 6f). This system provided detection of Pb^2+^ on the graphene FET surface with a LOD of 0.02 nM. Furthermore, the authors also reported an upper limit detection of 100 nM, which appeared to be larger than the maximum Pb^2+^ trace allowed in drinking water (72 nM). Wang et al. reported the use of 8-17 DNAzyme on graphene FET for Pb^2+^ detection in children’s blood [157]. Generally, as previously mentioned, the DNAzyme-based sensor is dependent on the cleave reaction as a signal for target detection. Nevertheless, in this case, the cofactor (Pb^2+^) was binding alone without cleaving the substrate strand. This is only possible when the research group replaces the RNA base (adenine) in the substrate strand with a DNA base. They intended to make the Pb^2+^ interact with the DNAzyme without cleaving the substrate strand. They reported the detection limit of Pb^2+^ as low as 37.5 ng/L with superior selectivity in competitor ions electrolyte and in real samples of children’s blood. In the same context of detection of metal ions using DNAzyme, Chang et al. demonstrated graphene FET sensor functionalized with mercury-dependent DNAzyme for recognition element for Hg^2+^ with a detection limit of 1 nM [158]. In this work, the authors utilized 2 nm-thick aluminum oxides (Al_2_O_3_) as passivation on the FET channel area to avoid ions intruding from analytes, and for the purpose of GFET sensor stability and accuracy. In order to discriminate Hg^2+^ in electrolyte, Hg^2+^-dependent DNAzyme was employed on the FET sensing channel. The detection mechanism of this sensor was based on the thymine–Hg–thymine (T-Hg-T) formation [159,160,161].

Besides the biological recognition probes presented in this review, there are many other effective sensing probes that could be used to detect HMIs on graphene surfaces. Other examples outlining the electrical detection of heavy metal ions on graphene FET are summarized in Table 4.

## 6. III-V Materials High Electron Mobility Transistor (HEMT)

Wide band-gap group III/V compounds, such as gallium nitride (GaN) and gallium arsenide (GaAs), are chemically stable semiconductors with high temperature/high power capability and high electron saturation velocity, which make them excellent materials for sensitive yet robust sensors. Aluminum gallium nitride (AlGaN) on a sapphire substrate is a great combination for the fabrication of high temperature sensors, as the thermal expansion coefficient of the sapphire substrate is in close proximity to Al_2_O_3_ or aluminum nitride (AlN) ceramides, which are often used as packaging materials for high temperature devices [170]. Both of the wide band-gap materials have been reported for the high thermal stability and long-term reliability, which is befitting for their use in extreme environments [171,172,173,174].

**Figure 9:**
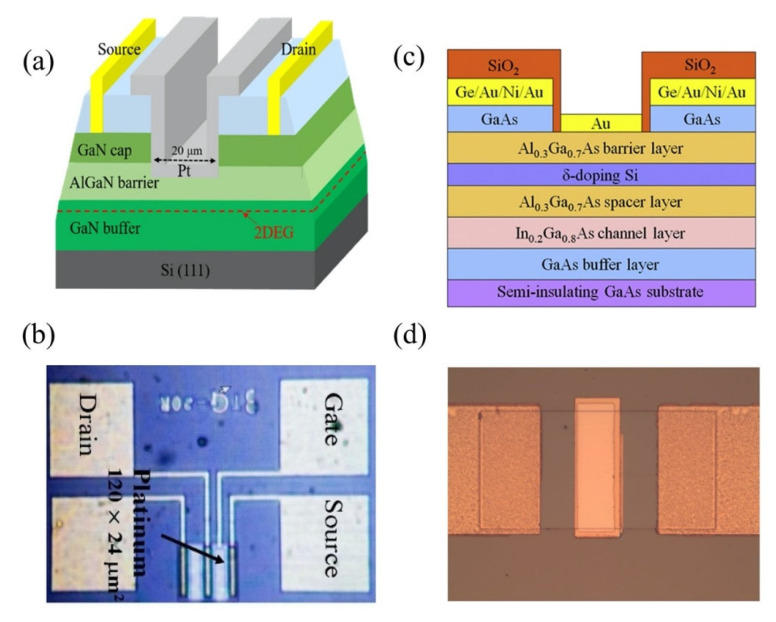
High electron mobility transistor devices. (**a**) A typical epitaxial structure of an Al-GaN/GaN HEMT sensor. (**b**) Top view image of an AlGaN/GaN HEMT sensor with floating gate. Reproduced with permission from [175]. Copyright 2021 MDPI. (**c**) AlGaAs/InGaAs HEMT structure. (**d**) Top view image of AlGaAs/InGaAs sensor proposed by Wang et.al. Reprinted from [182], Copyright 2015, with permission from Elsevier.

To date, the AlGaN/GaN high electron mobility transistor (HEMT) devices have been intensively studied for ion and biomolecule sensing applications such as gas sensors, chemical sensors and biosensors [175,176,177]. A typical HEMT epitaxial sensor device structure consists of a substrate (usually Si or sapphire), GaN buffer layer, AlGaN barrier layer, and GaN cap layer. Source and drain terminals were formed on the GaN cap layer, separated by a sensing area (gate). An example of a standard AlGaN/GaN HEMT three terminal device structure with a floating gate is shown in Figure 9a, and its top view microscopic image is in Figure 9b. Unlike conventional FETs in which channel conduction is governed by the majority carrier of holes or electrons (depends on the doping), HEMT’s conducting channel is fueled by two-dimensional electron gas (2DEG) arising from spontaneous piezoelectric polarization at the [0001] axis of the AlGaN/GaN heterostructure. Even though the 2DEG carrier mobility is incomparable to mobility of zero-bandgap graphene [178,179], HEMT has considerable potential as a highly sensitive sensor device with fast response time attributed to its higher 2DEG density and thinner barrier in the AlGaN/GaN layer, enabling direct detection of molecules or charged particles absorbed on top of its sensing area (gate) [180,181].

In recent years, some new sensing strategies for HMIs on AlGaN/GaN HEMT have been brought to light. For example, Nigam et al. and co-workers are currently one of the active groups working on AlGaN/GaN HEMT for the detection of heavy metal ions at the Indian Institute of Technology Jodhpur (India). The group of Nigam established an AlGaN/GaN HEMT functionalized with mercaptopropionic and glutathione (MPA-GSH) on the gate terminal for cadmium (Cd^2+^) ion identification [183]. GSH, a thiol-containing tripeptide, has been reported to be able to create binding with the Cd^2+^ ion [184,185]. This is supported by a published article, which reveals that the GSH-Cd binding forms a spherical shaped (GS)_4_Cd complex [186]. The MPA in this experiment served as a linkage between the Au covered terminal gate and glutathione. This sensor provided a LOD of 2.2685 nM and a sensitivity of 0.241 μA/ppb. Perhaps the most serious disadvantage of this method is the selectivity of the sensor. Even though the sensor demonstrated good selectivity towards Cd^2+^ ions, there were interferences from other heavy metal ions such as Cu^2+^, Hg^2+^ and Pb^2+^ in the selectivity analysis. It was reasonable to expect the sensor to respond to these HMIs because both Pb^2+^ and Hg^2+^ would form linear bonds with GSH, and the binding of Cu^2+^ by the MPA-GSH functionalized surface has been previously reported [187,188]. In the same year, Nigam and co-workers further reported a similar HEMT device functionalized with 2,5-Dimercapto-1,3,4-Thiadiazole (DMTD) for the detection of Pb^2+^ ions [189]. The detection of Pb^2+^ using DMTD ligand has previously been observed with different platforms [190,191]. Exploiting the similar idea, Nigam functionalized the DMTD ligands on the AlGaN/GaN HEMT gate terminal and observed its electrical response. The detection mechanism of this sensor was in a straightforward manner as the Pb^2+^ binds with the dangling thiol on the DMTD ligands, forming a Pb-DMTD complex. Due to this event, the density of 2DEG increased, which reflected the drain current increase. This investigation was more thorough than the previous one, as Nigam provided us with repeatability, recovery data and detection of Pb^2+^ in actual contaminated water, which is crucial to prove the practicality of the HEMT sensor in the real world. As disclosed by Nigam in his published article, the detection readings by his proposed HEMT DMTD-functionalized in as-prepared 48.826 nM Pb^2+^ ions electrolyte solution, tap water and lake water of Kaylana Lake (Jodhpur, India) are compatible with the readings of inductively coupled plasma mass spectroscopy (ICP-MS) that was used as a reference method in the investigation. The HEMT ion sensors achieved a sensitivity of 0.607 μA/ppb with a LOD of 86.87 pM. The sensor had rapid detection (response time approximately 4 s) and excellent repeatability, as only a small variation was observed when performing the sensing operations. Furthermore, the research group also demonstrated a new strategy for detecting mercury ions (Hg^2+^) using a molybdenum disulfide (MoS_2_) functionalized HEMT sensor [192], concerning the potential binding of the MoS_2_ with Hg^2+^ ions. The MoS_2_ consists of layers of one molybdenum sheet sandwiched between two sulfur sheets (S-Mo-S), which provide sulfide group layers as a site binding of Hg^2+^ ions. The authors reported sensor sensitivity of 0.64 μA/ppb with a detection limit of 57.43 pM and a response time of 1.8 s. 

**Figure 10:**
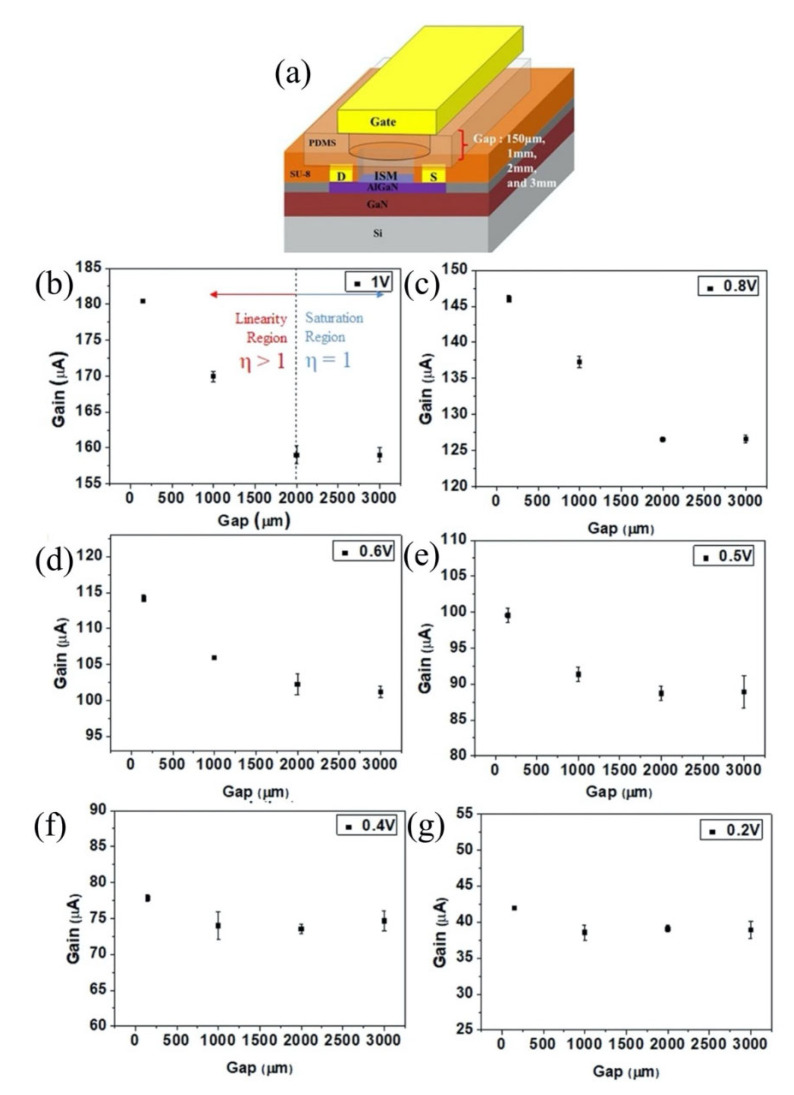
(**a**) Test setup to determine the effect of gate terminal distance and applied gate potential on sensor response (**b**–**g**) relationship of AlGaN/GaN HEMT current gain with different gate terminal distances and applied gate potential. Reprint with permission from reference [201]. Copyright 2018 Springer Nature.

Another popular HEMT sensing strategy for HMI detection was integration with an ion-selective membrane (ISM). A sensing membrane is generally comprised of a primary ion (ionophore) and an ion exchanger (lipophilic salt) incorporated into a polymeric membrane [193,194]. The ionophores are employed to selectively bind with target ions, as convincingly reported in various conditions, for instance, laboratory-prepared chemical analyte, environmental sampling, blood or serum [195,196,197,198,199]. Chen et al. and co-workers have demonstrated the detection of Pb^2+^ with a metal ion selective membrane (metal-ISM) in combination with AlGaN/GaN HEMT [200]. In this work, the author focused more on the HEMT configuration instead of modification of the sensing area to improve the sensitivity of the HEMT sensor. The research group successfully demonstrated the ultra-sensitive Pb-ISHEMT (lead-ions selective high electron mobility transistor) beyond Nernst response. They confirmed that the sensitivity of the HEMT depended on the distance of the gate terminal from the HEMT channel and the applied gate potential. Figure 10a depicts the AlGaN/GaN HEMT test setup for evaluating sensor response with the variation of gate terminal distance and applied gate potential. Their findings show that the shorter the distance between the channel and the gate terminal, the higher the electric field available to modulate the carrier density of the HEMT channel, and the higher the current gain yielded. Moreover, the applied gate potential also plays an important role in the sensitivity of the Pb-ISHEMT sensor. The low applied gate potential yielded small gain currents; higher gate potentials were necessary to obtain larger gain currents. The experiment data of the effect of terminal gate distance and applied gate potential on the sensor response is presented in Figure 10b–g. With the optimized device configuration and gate potential, the Pb-ISHEMT sensor is capable of discriminating Pb^2+^ as low as 0.1 nM. In another published article, Chen experimentally confirmed that the sensitivity of an ISM-based HEMT sensor was not only reliant on the site binding theory (which observed the number of site binding metal ions depending on the surface porosity and roughness of the ISM), but instead, the linear HEMT device operation contributed to sensor sensitivity beyond Nernst response [201]. Chen mentioned the significant relationship between gate terminal distance and applied gate potential, which renders an ultra-sensitive HEMT sensor, and the authors reported the detection limits of Pb^2+^ and Hg^2+^ were 0.1 nM and 0.01 nM, respectively. In a different study, Hsieh et al. also worked on the Pb-ISHEMT sensor and proved Chen’s hypothesis [202]. Similarly, the distance between the gate terminal and the channel area, as well as the applied potential gate have been taken into account in order to reproduce beyond Nernst’s ultra-sensitive sensing performance. The sensor produced a good linear response towards Pb^2+^ over a wide dynamic range concentration of 0.1 nM to 10 µM with a LOD of between 0.1 nM and 0.01 nM. The results were in a good agreement with Chen’s theoretical and experimental data. Although reporting excellent sensor performance (sensitivity beyond Nernst response, low LOD and wide detection range), the article lacks important performance analysis such as selectivity performance in mixed solutions, detection in real samples and reproducibility data to substantiate the practicality of this sensing method in the real world. 

Now we will turn to the mercury ion selective membrane (Hg-ISM) on AlGaN/GaN HEMT sensor for detection of Hg^2+^. Sukesan et al. constructed an array of Hg-ISHEMT sensors and connected them with a single FET that functioned as a switch to turn on each Hg-ISHEMT sensor [203]. The author reported an LOD and dynamic range of 0.1 pM and 0.1 pM–10 µM, respectively. The main driving force of this sensor, similar to the other ISM-based HEMT sensors, is based on the changes in dielectric capacitance (C_d_) when the Hg^2+^ binds with the ionophore. The electrolyte/semiconductor surface potential dropped as the Hg^2+^ concentration increased, leading to an increment in current [204,205]. It is worth mentioning that the sensor was independent to pH and conductivity, which means that the sensor could provide an accurate reading in real time. The research group had examined the sensor performance in real tap water samples and the findings demonstrated that it had a similar response to the data in mercury test solutions. Moreover, the sensor had good specificity towards Hg^2+^ even in complex matrices. Later, they constructed Hg-ISHEMT sensors with a user interface that could estimate the concentration of Hg^2+^ in an unknown solution [206]. Interestingly, the response time of this sensor was observed to be further improved up to 5 min, compared to previous Hg-ISHEMT sensor response time (~10 min). As could be expected, the sensor achieved a sensitivity of −36 mV/log, which surpasses ideal Nernst slope (−29 mV/log) and detection limit of 0.1 pM. Moreover, the sensor sensitivity and detection limit were found to be autonomous in high concentrations of interfering ions, implying that no pre-treatment or re-calibration is required when testing in various solution test samples. Integration of Hg-ISHEMT with the user interface expanded the possibility for commercialization. Other electrical detection of heavy metal ions on AlGaN/GaN HEMT sensors is summarized in Table 5.

As reported by several papers mentioned above, AlGaN/GaN HEMT is a popular platform for HMIs detection. On the other hand, AlGaAs/GaAs HEMT has been rarely reported as a biosensor in recent years, let alone for HMIs detection. In comparison to the AlGaAs/GaAs HEMT device, the AlGaN/GaN HEMT possesses a larger sheet carrier concentration at the surface owing to a larger piezoelectric effect, which consequently contributes to its popularity as a highly sensitive biosensor [207]. In the past ten years, there has only been one study published on HMIs detection using an AlGaAs/InGaAs HEMT sensor. Wang et al. conjugated one-end thiol modified ssDNA on the Au gate electrode of the AlGaAs/InGaAs HEMT device for specific detection of mercury (II) ions [182]. The AlGaAs/InGaAs HEMT structure and its top view photomicrograph in Wang’s work are shown in Figure 9c,d, respectively. This HEMT sensor sensing mechanism relies on the coordination interaction of Hg^2+^ with bis-thymine. The Hg^2+^ presence at the gate electrode changed the surface charges of the HEMT, resulting in the change of 2DEG concentration. The AlGaAs/InGaAs HEMT sensor successfully detected Hg^2+^ ions as low as 10 nM. This observation provides conclusive evidence that the AlGaAs/GaAs HEMT structure is suited for bio- and chemical sensing applications. However, the only drawback to this AlGaAs/InGaAs HEMT sensor is the complexity of the heteroepitaxial layer growth. 

## 7. Transition Metal Dichalcogenides (TMDs)-Based FETs

Graphene has successfully gained popularity as an electrode material for many applications at nanoscale sizes ranging from electronic devices to sensing and actuation. However, with zero bandgap characteristic, the application of graphene as a nanoelectronic device is limited. This graphene drawback has motivated the search for a more suitable material. Transition metal dichalcogenides (TMDs) materials are a promising candidate for nanoelectronics applications owing to their direct and tunable small bandgap properties. Being physically similar to graphene, the TMDs semiconductor materials have a large surface area and high carrier mobility that is well-suited for sensing applications [213]. Moreover, TMDs have become an attractive material for FET applications as they offer gate-tunable conductance owing to high current on/off ratios [214]. This attractive factor of high current on/off ratios plays an important role in sensor response time. The higher the current on/off ratios, the faster the response speed of the sensor [215]. 

**Figure 11:**
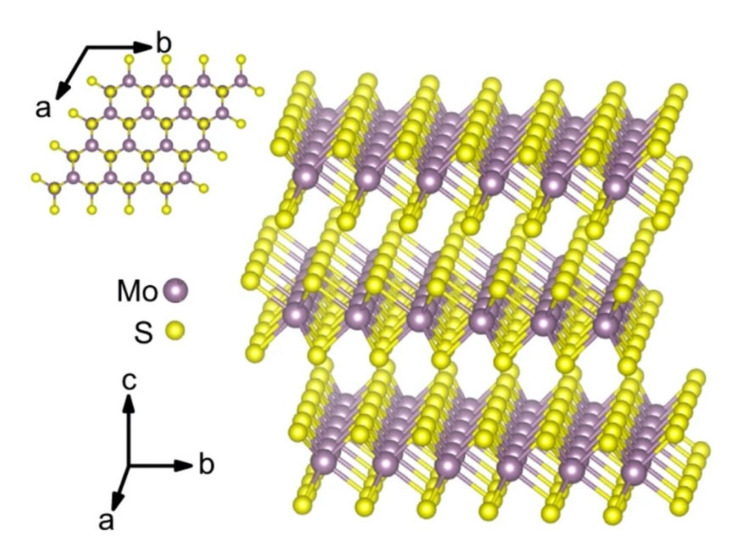
Atomic structure of a single layer of transition metal dichalcogenides (TMD). Reprint with permission from reference [217]. Copyright 2021 Springer Nature.

The TMD materials are a combination of transition metal elements with chalcogen atoms in which their structure can be defined as X-M-X or MX_2_ (M represents the metal transition while X represents the chalcogen atoms). Simply put, the TMDs are made up of a plane of transition metal that is sandwiched between two layers of chalcogen atoms. An illustration of TMDs’ atomic structure is given in Figure 11. The transition metal candidates include molybdenum (Mo), tungsten (W), titanium (Ti), zirconium (Zr) or Hafnium (Hf), whereas the flexibility of chalcogen element choice is rather limited. Three elements that are often found in the literature are sulfur (S), selenide (Se) and tellurium (Te). Since the seminal work by Dickinson and Pauling synthesized molybdenum disulfide (MoS_2_) in 1923, the S has been a popular choice of chalcogen atom of TMD material and has shown promise in evolution [216]. Se has been recently gaining popularity as an element in TMDs due to its high electrical conductivity, while Te is an expensive option to synthesize TMDs material; consequently, its potential is only suited for specific applications, making it the least popular option in the family of chalcogens. The application of TMDs as sensing and biosensing materials, began in 2013, which primarily focused on electrochemical fluorescence sensing. Therefore, the TMD material for sensing applications is still considered to be in its infancy stage. So far, only MoS_2_ and WS_2_ materials have been reported as FET materials for heavy metal ion detection. In the near future, we can expect the other TMD compounds to be exploited for FET sensing applications. 

MoS_2_ is the most intensely studied TMD material due to its synthesis maturity technology, stability and ruggedness as well as high availability [218]. The TMDs-based FET, pioneered by Radisavljevic et al. in 2011 [219] using MoS_2_ material as a conductive channel of a FET, paved the way for the development of the TMDs-based FET sensor. In recent years, increasing interest has been paid to TMDs materials as an FET platform for HMI detection. Zhou et al. demonstrated a MoS_2_-based FET for the detection of Hg^2+^ using DNA-AuNPs as probes to capture the ions [220]. The changes in MoS_2_ conductivity upon detection of Hg^2+^ can be explained by electron transfer from Au NPs to MoS_2_ film, resulting in a decreasing concentration of holes, which consequently induces its conductivity. This MoS_2_/DNA/Au-FET sensor demonstrated rapid response within 1–2 s with an ultralow detection limit of 0.1 nM. Notably, this sensor’s response time was faster than that of the electrochemical sensor, which takes 30 s to 180 s to respond [221,222]. One plausible explanation for the ultrasensitivity of this sensing strategy was attributed to MoS_2_. To verify this hypothesis, the research groups carried out a systematic series of experiments to investigate the relationship between sensing performance and different MoS_2_ thin film thickness, carrier mobility, and size of the bandgap (of MoS_2_). Their results indicated that a thinner MoS_2_ film with greater carrier mobility, and a wider band gap, would result in increased sensitivity (lower detection limit). Detection of Hg^2+^ on the bare MoS_2_ thin films has also been reported. Jiang et al. have investigated the performance of the MoS_2_-FET sensor in the detection of Hg^2+^ without any sensing probe [223]. In this study, Jiang et al. investigated the absorption of Hg^2+^ on a few-layers of the MoS_2_-FET channel. The findings of their studies revealed that, without Hg^2+^ presence, the MoS_2_-FET current-voltage curve reflected an n-type semiconductor characteristic. However, upon introducing Hg^2+^ to the FET channel, the drain current decreased. Furthermore, the authors claimed that as the Hg^2+^ concentrations increased, the MoS_2_-FET current-voltage characteristics progressively changed to p-type. This phenomenon suggests that the coordination of Hg^2+^ with the S^2-^ ligands on the surface of MoS_2_ leads to a p-type doping effect due to partial electron transfer from MoS_2_ to Hg^2+^. On this basis, they further investigated the p-type doping effect of Hg^2+^ on the MoS_2_ surface using photoluminescence spectroscopy. The result of photoluminescence studies were in line with the FET measurement. This sensing strategy yielded a detection limit as low as 30 pM.

**Figure 12:**
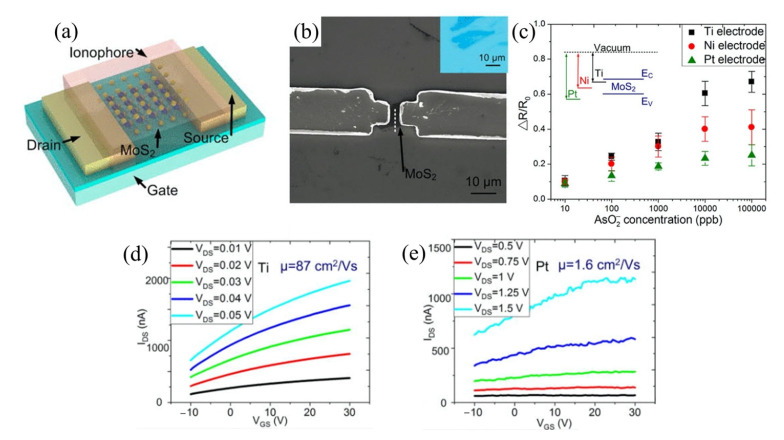
Relation of Schottky barrier height with sensitivity of MoS_2_ FET sensor. (**a**) Schematic diagram of MoS_2_ FET sensor with ionophore thin film. (**b**) An optical image of MoS_2_ flake (in inset) and top view of MoS_2_ FET sensor device. (**c**) Relative resistance of MoS_2_ FET sensor in different AsNO_2_^-^ concentrations with different types of metal contact electrodes (titanium (Ti), nickel (Ni) and platinum (Pt). Titanium metal contacts show the best sensitivity. (**d**) Drain current of MoS_2_ FET with titanium metal contacts and (**e**) drain current of MoS_2_ FET with platinum metal contacts. Reprinted from [224], with the permission of AIP Publishing.

Li et al. and colleagues demonstrated MoS_2_-FET with different metal contacts for detection of arsenic [224]. They utilized an ionophore thin film to selectively permeate arsenic dioxide (AsO_2_^-^) onto the MoS_2_ FET channel and serve as a negative gate voltage. When the AsO_2_^-^ ions bound to the MoS_2_ surface, the electrons in the bulk were repelled (pristine MoS_2_ is an n-type semiconductor in nature), resulting in lower MoS_2_ conductivity. The change in conductance was expected due to the absorption of the target molecules on the MoS_2_ surface [225]. A schematic diagram of MoS_2_-FET with an ionophore thin film and its top view optical image are shown in Figure 12a,b, respectively. Further, the research group investigated the Schottky barrier (SB) height at the metal contacts of the source/drain in relation to the sensitivity of the MoS_2_-FET sensor in an aqueous environment, inspired by the work of Liu et al. who investigated the sensing performance of the MoS_2_ gas sensor with a different height of Schottky barrier [226]. The sensitivity of the MoS2-FET towards AsO_2_^−^ was examined using three different metal contact work functions (Φ), namely: platinum (Φ_Pt_ = 5.9 eV), Nickel (Φ_Ni_ = 5.0 eV) and titanium (Φ_Ti_ = 4.3 eV). The relationship of Schottky barrier height with the work function of the metal contact can be described as Φ_SB_ = Φ − Ψ, where Ψ is the MoS_2_ electron affinity constant. Therefore, the Schottky barrier height of MoS_2_ FET can be placed in the order of Ti < Ni < Pt according to their work function. Experimental results in Figure 12c show that the shortest Schottky barrier height (titanium) demonstrated the best sensitivity as the FET device showed the biggest resistive change when operating in an aqueous environment. Beyond that, the MoS_2_ FET with a shorter Schottky barrier height determined better device conductivity. Figure 12d,e present a comparison of FET drain current performance with titanium and platinum metal contacts. From the results, the FET with titanium metal contacts has better conductivity (higher drain current) compared to the FET with platinum metal contacts. This study has shown that the reduction of Schottky barrier height has conclusively improved the MoS_2_ sensor performance. This sensor was reported to be able to detect As^3+^ as low as 1.3348 nM with excellent selectivity towards AsO_2_^−^, owing to the ionophore film to filter out other interference ions. Recently, Li and his research group further explored their interest in the MoS_2_-FET sensor by investigating the influence of tensile strain on the sensing performance of flexible MoS_2_-FET ion sensors [227]. Strain is a critical parameter for flexible/stretchable chemical devices. Several studies have concluded that the strain could create a piezoresistive effect on flexible MoS_2_ material, which the resistivity of the material changes due to band structure changes during mechanical deformation [228,229,230]. The results of the studies showed that the drain current of the FET increased as the strain was increased from −0.17% to 0.17%, suggesting the tensile/compression strain promoted the band gap reduction and consequently enhanced the electrical conductivity. This MoS_2_-FET ion sensor behavior under strain conditions shows excellent agreement with the experimental results of a recent study by John et al. [231]. For sensing studies, the MoS_2_-FET ion sensor was fabricated in an array and functionalized with four different ionophores (sodium ionophore, cadmium ionophore, mercury ionophore and lead ionophore). The resulting sensor exhibited merit for simultaneous detection with exceptional selectivity towards sodium, cadmium, mercury and lead ions. Under a strain condition, the MoS_2_-FET ion sensor was able to detect Cd^2+^ as low as 5 ng/mL with a response time of 8 s. In addition, the sensor demonstrated promising potential in real-life applications as it successfully monitored Hg^2+^ ions in tap water and Na^2+^ ions in human sweat samples. Next, MoS_2_ material was also reported in the fabrication of the chemiresistor ion sensor. A recent published study by Bazylewski et al. [232] demonstrated that MoS_2_-chemiresistor functionalized with L-cysteine (Cys) exclusively for the detection of Cd^2+^. The sensor devices were reported to be able to detect Cd^2+^ ions in the range of 8.896 nM–4.448 µM at neutral pH with a response time of ~1 s. The studies also explored the effect of pH on the chemiresistor performance. The pH-dependent experiment was carried out in buffered solutions pH 3–10. The results revealed that the sensor struggled with selectivity at lowest pH (<pH 5) and highest pH (>pH 9), limiting the detection window to pH 5–9. It is worth noting that the detection in pH 6–7 demonstrated high selectivity towards Cd^2+^ despite the presence of other interference metal ions with similar hydrodynamic radii. The resistivity of the chemiresistive sensor also increased by 20 times in water that contained a 44.48 nM concentration of Cd^2+^ ions.

**Figure 13:**
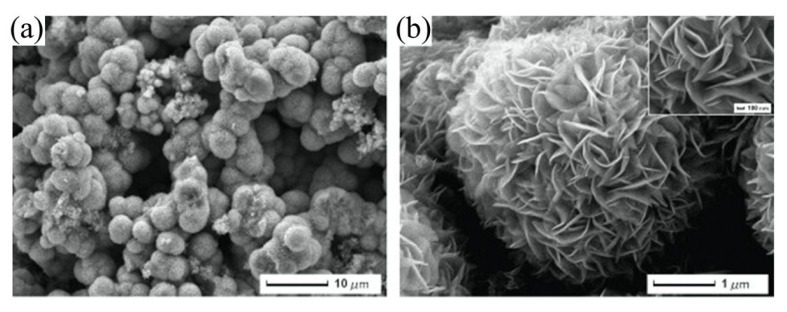
SEM images of 3-D flower like MoS_2_ morphology. (**a**) flower-like MoS_2_ composed of a large quantity of uniform MoS_2_ microspheres. (**b**) Magnified view of MoS2 microsphere. A MoS_2_ is built from several dozens of nanosheets that connected through the center to form 3-D flower-like structures. Reprinted from [234], Copyright 2015, with permission from Elsevier.

A three-dimensional (3-D) flower-like MoS_2_ nanostructure for HMI detection has also been reported. As shown in Figure 13a,b, the 3-D flower-like MoS_2_ is made up of a large quantity of MoS_2_ microspheres, each of which is built from nanosheets that tie together at the center to form a 3-D flower-like structure. The unique structure of MoS_2_ flower-like has attracted a great deal of attention in nano sensor applications. It has the major advantage of maximizing the performance of the sensor, owing to its high surface area, which provides large numbers of potential reaction sites. An et al. developed a 3-D MoS_2_-based FET aptasensor to detect Arsenic (III) in real river water [233]. In this work, the sensor was fabricated as an interdigitated microelectrodes array (IDA) on a glass substrate. The surface of the glass substrate was treated with 3-aminopropyltrimethoxysilane (APS), to provide amino functional groups as anchors to carboxylic polypyrrole (CPPy)-coated flower-like MoS_2_ nanospheres (CFMNSs). Finally, arsenic binding aptamer (Ars-3) was immobilized on top of the CFMNSs as a probe to recognize As^3+^. The resulting sensor could detect As^3+^ as low as 1 pM, and the sensor response in less than 1 s. The response time of the 3-D flower-like MoS_2_ FET aptasensor was faster compared to the 3-D flower-like MoS_2_ functionalized AlGaN/GaN HEMT reported by Nigam et al. which responded on a time scale of 1.8 s [192]. Moreover, the 3-D flower-like MoS_2_ FET aptasensor had excellent selectivity towards As^3+^ in real river water samples. The extraordinary performance of this sensor might be contributed by the CPPy coating layer, which enhances the conductivity of the MoS_2_ and the number of functional groups.

Besides MoS_2_, tungsten dichalcogenides are also receiving attention from researchers for their application in HMI detection. Neog et al. first described the potential of WS_2_ as a chemiresistor sensor for the detection of HMIs [235]. The result of his studies showed that the I-V characteristic of the untreated (bare) WS_2_-chemiresistor was linear in the voltage range of −9 V to 9 V and the maximum current obtained was in *n*A scale. The linear I-V characteristic reflects WS_2_ ohmic behavior, where the current flow is proportional to the direct voltage across the chemiresistive device. However, with the presence of 17.906 µM of Fe^3+^ and 16.968 µM Co^2+^ in the test solutions, the I-V characteristics of the devices were abruptly changed and the current was raised to µA range. Evidently, the WS_2_ chemiresistor device was no longer following Ohmic’s law. One unanticipated finding was that the reversible properties of WS_2_ nanosheet. When all the ions were removed from the WS_2_ surface, the chemiresistor restored the original electrical behavior. This finding opens the opportunity for the WS_2_ chemiresistor as a reusable electrical detection for heavy metal sensors. Despite this promising result, repeatability and reproducibility studies of the WS_2_ chemiresistor devices are recommended to determine its stability and reliability. The LOD of this WS_2_ chemiresistor was 30 µL to change its electrical behavior to atypical (non-ohmic). In another study, Neog et al. further explored the potential of the WS_2_ chemiresistor by detecting different HMIs [236]. The WS_2_ chemiresistor exhibited resistance changes towards eight different ions (As^3+^, Ni^2+^, Cu^2+^, Sn^2+^, Se^4+^, Hg^2+^, Pb^2+^ and Zn^2+^), but the most prominent resistance changes of the WS_2_ chemiresistor occurred in the presence of Zn^2+^. Later, the Zn^2+^ content present in the solution was quantified by using the peak and valley difference method. The WS_2_ chemiresistor showed high selectivity to Zn^2+^ ions, with a LOD of 14.375 ± 0.7646 nM, and a sensitivity of 0.63 ± 0.05 µA/ppb.

## 8. Other FETs

### 8.1. Black Phosphorus FET

Black phosphorus (BP) is one of the members of a two-dimensional (2D) family of materials that possess unique properties, including biocompatibility, in-vivo biodegradability, large surface-to-volume ratio and tunable bandgap [237]. BP’s electronic properties are superior to those of other semiconductor materials. BPFET is reported to have higher carrier mobility (1000 cm^2^.V^−1^.s^−1^) than TMD-FET [238]. Moreover, the BP current on/off ratio (10^3^–10^5^) is larger than published graphene transistors [239,240]. On top of that, BP’s molecule absorption energies are larger than those of graphene and MoS_2_ [241]. Despite these remarkable properties, BP appears to be unstable in ambient conditions [242,243,244]. As a result, it makes BP less attractive for practical application. In recent years, many researchers have attempted to improve BP stability, thereby enabling its potential for biosensor application. Firstly, the implementation of passivation has been proven as a possible approach to stabilize the black phosphorus in the air. Upon passivation, different materials have been suggested to be incorporated onto the BPFET sensing channel, aiming to improve stability. For example, Li et al. reported [245] the use of lead ionophore as passivation on their BPFET. The specified ionophore not only improves the stability of BP, but it also serves as a sensing element to detect a specified ion. An ionophore is a coating film that can discard undesired ions from the environment and only allow specified ions to permeate through it. In this case, only Pb^2+^ was expected to reach the BP sensing area while other ions are filtered through the ionophore film. The Pb^2+^ that permeates through the ionophore film functions as a positive gate voltage and, consequently, repels the positively charged holes in the channel area, leading to conductance decrease. The BPFET sensor shows an LOD of 4.826 nM. Another good passivation suggested to promote BP stability is SiO_2_, Al_2_O_3_, aryl diazonium, titanium sulfonate ligands and PEG [246,247].

Dithiothreitol (DTT) conjugated with AuNPs was functionalized on the BPFET for the detection of As^3+^ in water [248]. In addition to functioning as an As^3+^ recognition element, DTT is also well known as a reducing agent of As^5+^ to As^3+^ [249,250,251], suggesting that the proposed BPFET sensor would be able to detect As^5+^ as well. The detection of this sensor was based on the simple interaction of thiol-containing ligands at DTT with As^3+^ [4]. A detection limit of 1 nM was obtained, and this ultrarapid sensor demonstrated a fast response of 1–2 s. Since thiol is a versatile probe for many metal ions, the selectivity of the sensor was a bit complicated. Hence, ethylenediaminetetraacetate (EDTA) was added into the test solution during selectivity analysis to suppress the activities of other metal ions [252], resulting in a significantly improved specificity of the sensor towards As^3+^.

**Figure 14:**
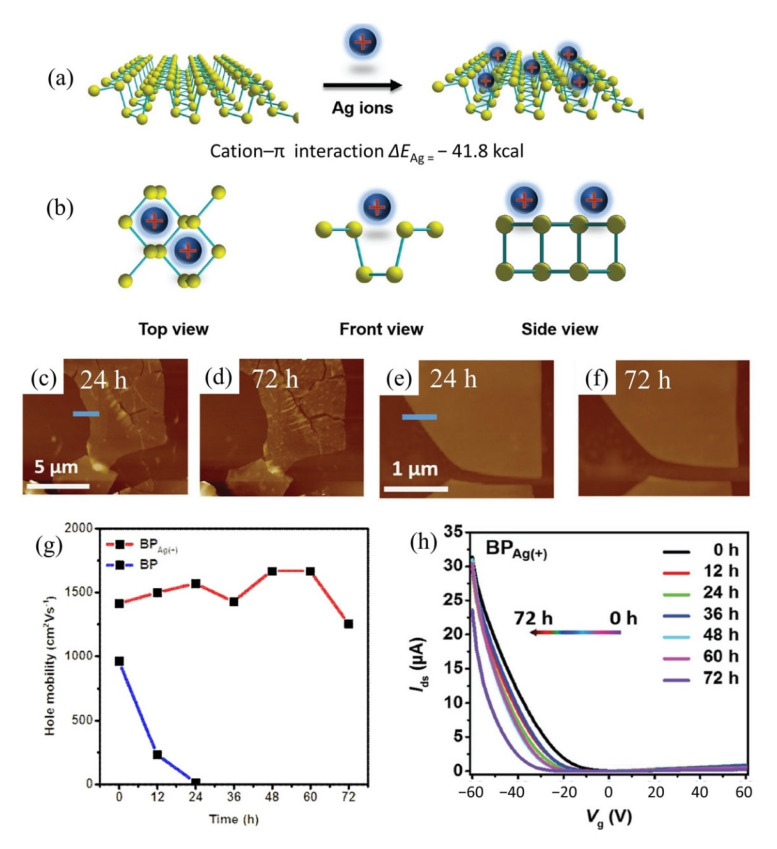
(**a**) Absorption of Ag^+^ on BP layer through conjugated π bond. (**b**) Possible views absorbed Ag^+^ on BP layer. (**c**,**d**) AFM images of bare BP exposed to air day 1 and day 3, respectively (**e**,**f**). AFM images of BP_Ag+_ exposed to air day 1 and day 3, respectively. (**g**) Holes’ mobility of BP and BP_Ag+_ up to 3 days. (**h**) Drain current performance of BP_Ag+_ up to 72 h. Reprinted with permission from reference [255]. Copyright 2017 John Wiley and Sons.

The direct detection of metal ions on the BP without any surface functionalization or passivation has also been reported. Wang et al. described a simple approach for electrical detection of Ag^+^ using black phosphorus FET (BPFET) [253]. The sensing mechanism of this sensor relies on the direct absorption of Ag^+^ on the BPFET sensing surface. The HMIs Ag^+^ interacts directly with the conjugated π bond derived by the lone pairs of phosphorus atom in the surface layer as illustrated in Figure 14a. There are three different possible views of the Ag^+^ absorption on the BP layer shown in Figure 14b. This sensor could detect Ag^+^ at a detection limit of 10^−10^ mol/L (92.7 pM), which was lower than the safe limit of Ag^+^ in drinking water set by the World Health Organization (0.927 µM). The response of this sensor was averaged at 60 s for each sample. While the sensor yielded good sensitivity towards single ion detection of Ag^+^, the selectivity of this BPFET sensor in multi-ions electrolyte was unknown. Evidence shows that the conjugated π bond on the phosphorus layer can also bind with other metal ions (Mg^2+^, Fe^3+^ and Hg^2+^) [254]. Therefore, an extended study to determine the sensor selectivity towards Ag^+^ in complex matrices is needed. It is also worth pointing out that the absorption of Ag^+^ could stabilize the BP layer. Guo et al. carried out a series of experiments to investigate the stability of BP material with Ag^+^ absorption [255]. Figure 14c–f compares the atmospheric stability of bare BP and BP_Ag+_ from day 1 to day 3, consecutively. It was observed that there were bubbles formed on bare BP after 24 h exposed to the atmosphere, and after day 3, the bubbles increased in size. Whereas in the case of BP_Ag+_, the surface layer was preserved and showed no obvious bubble formation. The hole mobility of BP_Ag+_ was also stable (1500 cm^2^Vs^−1^) at least up to 72 h when compared to bare BP, which degraded to 0 cm^2^Vs^−1^ after 24 h, as demonstrated in Figure 14g. The drain current of a BP_Ag+_-FET was degraded by 28% only after 72 h (Figure 14h). These multiple analyses revealed that the absorption of Ag^+^ on the BP surface layer promotes the stability of BP materials.

Chang et al. and colleagues introduced a semi-quantitative FET model to estimate and describe the direct absorption metal ion density, LOD, and sensing mechanism on the FET channel surface [256]. In this work, the research group developed a BP nanosheet FET sensor to validate the FET model. Based on ion concentration and intrinsic FET material properties, such as band gap and carrier density, they developed a statistical thermodynamics model that will be able to predict the LOD of a FET sensor. On top of that, the semi-quantitative model could also describe the sensing mechanism of the FET by relying on the relative size of the Debye length and the distance between absorbed ions. From the findings, they concluded the detection mechanism at low ion adsorption density was dominated by the charge transfer effect. While at high ion adsorption density, the detection of metal ions was predicted to be controlled by a gating effect. Application of the semi-quantitative FET model was evaluated in Na^+^, Mg^2+^, Zn^2+^, Cd^2+^, Pb^2+^ and Hg^2+^ solutions. Among the other metal ions, Hg^2+^ was found to be more responsive with this BP-FET sensor. The LOD of Hg^2+^ is predicted to be 0.1 nM in tap water and 0.1 fM in deionized water. The implication of this study raises the possibility that the BP-FET can be used as a probe-free detection sensor in a wide range of ion sensing applications.

### 8.2. Organic Field-Effect Transistor (OFET)

The organic field-effect transistor (OFET) belongs to the thin film transistor (TFT) family, which employs organic semiconductors (OSCs) as an active electrode. The OSCs are flexible, unbreakable, lightweight and have a low power consumption, which makes them an ideal electrode for TFT applications [257]. Many mistake OFET as an MOSFET. However, OFET is different than MOSFET in several ways. First, the principal operation of the OFET is rather straightforward compared to the MOSFET. OFET operates under accumulation mode [258], for which its conductance is directly controlled by the gate voltage applied. Secondly, the mobility of OSC is often lower (10^−1^~10^−2^ cm^2^/Vs^−1^) than that of crystalline silicon. OFET has been extensively used for biosensors, but only a few studies have reported on OFETs for heavy metal detection. Herein, we will discuss some of the recently developed OFETs for determination of HMIs.

Minami et al. developed an OFET sensor for detection of Hg^2+^ in sea water [259]. In this sensor, L-cysteine, which can selectively bind to Hg^2+^ ions through an Hg–S bond, was conjugated onto an extended-gate gold electrode as a recognition element. The main driving force of this sensor was laid on the interaction of Hg^2+^ with cysteine to form an Hg–Cys complex, which consequently, changed the OFET conductance. Even though the OFET sensor confirmed its sensitivity and provides a detection limit (LOD) of 154.54 nM, the performance of this sensor did not meet the expectations for measurement in real sea water samples. This is because the mercury level in the sea water sample is comparatively low (below 0.997 nM). Therefore, a sensor with better LOD is anticipated. It is worth mentioning here that the OFET sensor exhibits good selectivity towards Hg^2+^ ions, which is attributed to the high binding affinity of thiols in the L-cysteine structure for Hg^2+^. In another approach, an artificial receptor was used at the gate electrode of OFET to detect Hg^2+^ [260]. The extended-gate gold electrode was functionalized with thiolated DPA self-assembled monolayer (SAM) to probe Hg^2+^ in a prepared solution with the interference of Na^+^. Similar to the work described previously, the detection mechanism of the sensor was also based on the affinity binding of thiols with Hg^2+^. This method has achieved a detection limit of 49.35 nM, which is lower or comparable to the other sensors [261,262,263]. Further, the sensor was tested in a multi-ion electrolyte to evaluate its selectivity towards Hg^2+^. The findings reported that all the other interference ions’ responses are almost negligible, suggesting the sensor’s excellent selectivity. In this work, the reusability of the sensor has also been investigated. Surprisingly, the sensors exhibit no degradation after a few washes with an EDTA solution. In practical application, the reusability of a sensor is important for cost effectiveness.

Knopfmacher et al. used a high mobility polyisoindigo-based polymer with siloxane-containing solubilizing chains (PII2T-Si) as an active semiconductor material to efficiently detect Hg^2+^ in the marine environment [264]. In order to selectively detect Hg^2+^, the PII2T-Si surface was functionalized with DNA conjugated gold nanoparticles (AuNPs) prior to sensing in sea water. The DNA-AuNPs probes formed a hairpin structure upon binding with the Hg^2+^. This conformational change resulted in a negatively charged increase on the OFET’s surface, consequently resulting in hole carrier accumulation in the organic semiconductor. This sensing method allowed for an Hg^2+^ detection limit down to 10 μM. The most striking finding to emerge from this experiment is that the OFET sensor has the ability to detect Hg^2+^ in a high concentration of salt in sea water (~600 mM) without any sample pre-treatment. To the best of our knowledge, this is the first selective sensing strategy using polymer OFET in the marine environment. In practice, the OFET sensor easily deteriorates when in contact with aqueous media alongside high operating voltages. The instability of the organic semiconductor has restricted the application of OFET as a reproducible and reliable chemical or biological sensor in the real world. To overcome this shortcoming in OFET, recently, Sayyad et al. reported the incorporation of carbon nanomaterial in organic semiconductor [265]. In the analysis, reduced graphene oxide (rGO) was incorporated into the PEDOT: PSS to form a new hybrid material, the PEDOT: PSS/rGO nanocomposite, which has improved structural, morphological and electrical properties. Later, the PEDOT: PSS/rGO was utilized to fabricate an OFET for detection of Hg^2+^. PEDOT: PSS/rGO OFET exhibited a good linear response towards mercury ions over a wide range of concentrations, from 1 nM to 60 nM, with a LOD of 2.4 nM. In another study, Hg^2+^ detection was realized using polyaniline nanowires (PANI NWs) OFET functionalized with EDTA [266]. In the presence of Hg^2+^, the drain current was observed to be increased, indicating Hg-EDTA complex formation on the OFET surface. The OFET sensor sensitivity of 0.766 mA/ppb was obtained and the LOD of the sensor was down to 0.7214 nM.

**Figure 15:**
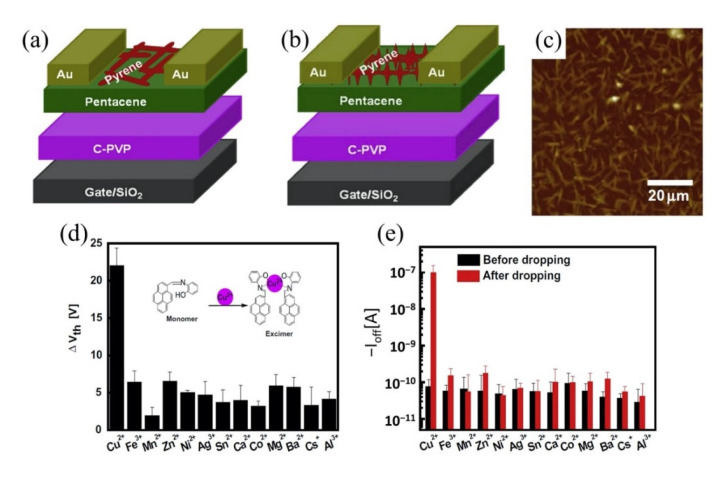
(**a**) Structure of OFET device functionalized with pyrene derivative. (**b**) Self-assembly of star-shaped pyrene derivatives in the presence of Cu^2+^. (**c**) AFM image of self-assembled star-shaped pyrene derivatives. (**d**) FET voltage threshold (V_TH_) change in the presence of Cu^2+^. (**e**) Off current change in the presence of Cu^2+^. Reprinted from [268], Copyright 2014, with permission from Elsevier.

In order to detect copper (II) ions in water, Sasaki et al. [267] proposed an unlabeled extended-gate OFET sensor decorated with nitrilotriacetic acid (NTA) monolayer. This simple approach achieved an LOD of 1.5106 µM for Cu^2+^ detection. The sensing mechanism of this sensor relies on the interaction of Cu^2+^ with NTA, which has induced a decrease in FET channel conductance. In selectivity analysis, the sensor showed no response to the other metal ions, however there had been a little response to the presence of Ni^2+^. The Ni^2+^ might be competing with Cu^2+^ to electrostatically bind with the receptor. Thus, the use of a masking agent to seclude the Ni^2+^ prior to sensing is essential to improve the sensitivity and selectivity of the sensor. In a related study, Ramesh et al. demonstrated the determination of Cu^2+^ using a pentacene/Schiff base pyrene derivative that would change to star-shaped in the presence of Cu^2+^ [268]. Figure 15a,b illustrates the before and after detection of Cu^2+^ on OFET functionalized with pyrene derivatives, respectively. The mechanism of the sensor mainly relies on the formation of excimer pyrene (P−P*). Chemically, upon the presence of Cu^2+^, monomer OH- functional group was deprotonated to let the hetero atoms (O and N) and Cu^2+^ (chelator) engage in the formation of an excimer P−P* [269], thereby leading to the V_TH_ and off current change. At the same time, the non-uniform pyrene thick rods on the OFET surface were broken into pieces and re-assembled into star shapes, as shown by the AFM image in Figure 15c. The OFET sensor demonstrated exceptionally good selectivity and sensitivity towards copper ions as the V_TH_ and off current changed significantly in the presence of Cu^2+^ as seen in Figure 15d,e, respectively. This approach yielded sensitivity in the range of 20–350 μM and a LOD of 50 μM.

### 8.3. Inorganic-Based FETs

Among the metal oxides (inorganic semiconductors), zinc (Zn), indium (In), gallium (Ga) and tin (Sn) are the most studied materials for electronic applications because they are non-toxic [270]. Binary compounds of these elements, such as SnO_2_, ZnO, In_2_O_3_ and Ga_2_O_3,_ have been employed as an active layer of TFTs [271,272,273,274]. Contrary to organic semiconductors, amorphous metal oxide or inorganic semiconductor materials have not received much attention in the sensor field, primarily due to their mechanical instability and mediocre performance compared to vacuum-processed inorganic devices [275,276]. Additionally, the oxide metal formation by the sol–gel technique requires a high processing temperature in order to obtain optimal electrical performance [277]. Metal oxide binary compounds have also been reported to have poor device performance (e.g., high electrical resistivity, unstable and low on-off ratio) [278], limiting their use in electronic device applications. Despite these drawbacks, metal oxide semiconductors do have notable advantages for sensing transducers, such as straightforward device fabrication, large surface area and fast deposition [279]. Therefore, many researchers have focused on these advantages for next-generation sensing devices. The present studies show that the metal oxides can still be feasible in sensing applications. We follow with a review the recent work on the metal oxides TFT specifically for heavy metal detection.

**Figure 16:**
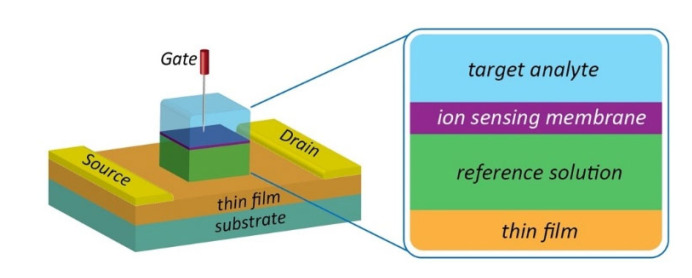
Illustration of twin pool gating FET setup.

Alqahtani et al. investigated SnO_2_ water-gated TFT (SnO_2_ WGTFT) to detect Pb^2+^ and Cu^2+^ [280]. In this investigation, the research group has employed natural zeolite clinoptilolite ionophore as a sensing element in a polyvinylchloride (PVC) membrane. The configuration of the SnO_2_ WGTFT sensor device was described as a twin-pool gating setup, which consists of two pools of tap water separated by a PVC based ion-selective membrane, inspired by the classical potentiometric ion sensor. The tap water at the bottom pool was as drawn (without added ions and functioning as a reference solution) and directly in contact with the SnO_2_ substrate, while the latter was added with a known concentration of lead or copper ions (target analytes). A tungsten (W) needle was immersed in the latter pool, acting as a reference electrode. For a proper understanding of this sensor configuration, we illustrated a cross-section of this TFT sensor in Figure 16. The sensing mechanism of the TFT sensor relies on the ion movement on the target/membrane interface, resulting in a potential difference between target analytes and reference solution, observed with the threshold voltage, V_TH_ shift. If the ion activities in the target analytes match those in the reference electrolyte, no V_TH_ shift will be observed. This potentiometric-TFT ion sensor offers several advantages over typical TFT. For example, it can be manipulated to sense various ions by introducing an appropriate sensing membrane. Clearly, this type of sensor does not require direct modification to the semiconductor TFT channel, which provides stability for the overall device. Moreover, since the TFT semiconductor channel does not involve any probe binding (e.g., aptamer or proteins), the potentiometric-TFT sensor allows for a simple recovery process. The sensitivity and detection limit of the TFT sensor can also be tuned by altering the properties of the sensing membrane [281]. This SNO_2_ WGTFT sensing strategy provided Pb^2+^ and Cu^2+^ detection with LOD of 0.9 nM and 14 nM, respectively. In another study of metal oxide based TFT for HMIs detection, Kim et al. utilized nanoglobules of ZnO/GO and ZnO/rGO for the development of multiple ion field-effect transistors (MI-FETs) sensor [282]. The sensitivity of the MI-FETs sensor was examined for a variety of ions, including Ni^2+^, Co^2+^, Cu^2+^, Cr^3+^, Fe^2+^ and Bi^2+^. However, the ZnO/GO and ZnO/rGO MI-FET only showed significant response towards Cr^3+^ and Cu^2^ for which the sensitivity was 49.28 mA µM^−1^·cm^−2^ and 185.32 mA µM^−1^·cm^−2^, respectively. The MI-FET nanoglobules ZnO/GO channel exhibited an LOD of 7.05 µM while the LOD of nanoglobules ZnO/rGO MI-FET was 14.9 µM.

Indium (III) oxide (In_2_O_3_) and tin-doped indium oxide (ITO) have been addressed as an interested field recently [283,284,285]. Both the In_2_O_3_ and ITO materials exhibit metal-like behaviors. ITO belongs to the transparent conducting oxides (TCOs). Its carrier density can be up to 10^21^ cm^−3^ and resistivity can be down to 10^−5^ Ω cm. The use of In_2_O_3_ and ITO as HMI FET sensors has not been investigated. However, ITO has been reported to be used in the OFET structure as a supporting material to detect Hg^2+^ [286]. ITO thin film served as the bottom channel material to increase the channel carrier concentration and maximum channel current of the OFET sensor. Cong et al. earlier used ITO in their TFT structure for similar purposes [287]. The OFET sensor in this work was conjugated with pyrene, which has been widely employed as recognition probes for mercury (II) ions owing to strong affinity binding between pyrene-thiol functional groups towards Hg^2+^. The LOD of the TFT sensor was observed to be 25 µM in tap, drinking and sea water samples. The response of the TFT sensor was found to be linear with the concentration of Hg^2+^ in the range of 1 mM to 0.01 µM.

**Figure 17:**
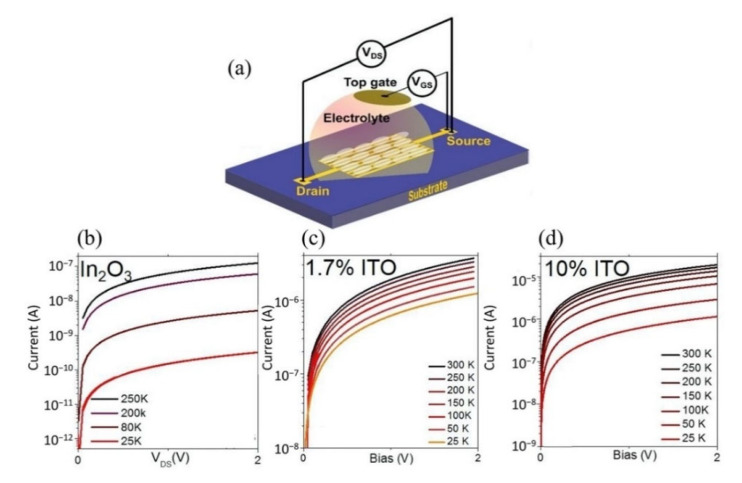
(**a**) Structure of ITO and In2O3 electrolyte gated FET. (**b**–**d**) FET characteristics of In_2_O_3_ and ITO (with 1.7% and 10% of Sn doping) under different temperatures. Reprinted (adapted) with permission from [242]. Copyright 2019 American Chemical Society.

A recent study by Qu et al. has unveiled the potential of ITO and In_2_O_3_ as transducer material for electrolyte gate FET [288]. In this study, Qu et al. developed ITO and In_2_O_3_ electrolyte gated FETs (Figure 17a) and studied their electrical behavior. In_2_O_3_ thin film and ITO (with Sn doping of 1.7% and 10%) have demonstrated FET current-voltage characteristics, as shown in Figure 17b–d. We noticed that the FET behavior was evaluated under different temperatures, suggesting the robustness of these thin films as FET sensor materials. Furthermore, Qu’s study also discussed the ligand binding options on these thin films, which established our understanding of ITO and In_2_O_3_ surface functionalization. According to Qu, hard acids such as acetic acid are more suitable as ligands for these indium based oxide materials compared to thiols because the hard base nature of In^3+^ has poor affinity towards thiol ligands [289]. These findings not only extend the application of ITO and In_2_O_3_ as potential materials for FET devices, but also stimulate new opportunities for investigating these thin films for HMIs detection.

## 9. Conclusions and Future Scopes

In the past 10 years, a significant effort has been made to realize high sensitivity and specificity of electrical detection for heavy metal ions (HMIs). Nanomaterial silicon, CNT, graphene, AlGaN/GaN, TMD, black phosphorus, organic and inorganic semiconductors have been demonstrated as a powerful and effective sensing platform for HMI detection in combination with different types of molecular recognition. At the same time, many detection strategies were explored on the FET platform to achieve ultrasensitive sensor performance. The lowest LOD obtained so far is as low as 10 fM, and most of the detection was achieved in real time. Within the past 10 years, graphene FET and AlGaN/GaN HEMT have rapidly shown an advancement in monitoring HMIs compared to other semiconductor nanomaterials. We believe that because of graphene’s versatility and availability, it has emerged as the preferred transducer material for FET sensors. Contrary to the AlGaN/GaN material, even though it is slightly expensive and has a complex fabrication process compared to the other materials, it nevertheless offers high sensitivity detection in high concentration solutions, and it has been proven that AlGaN/GaN HEMT can operate beyond the Debye length in physiological solutions (i.e., human serum). 

Despite this progress, the FET sensing method still has room for improvement. It offers a satisfied sensing platform for monitoring different kinds of HMIs. However, there are a few concerns that need to be addressed before an FET-based sensor can be realized commercially for HMI detection. So far, the majority of the works reviewed here have only been demonstrated as proof-of-concept sensors capable of detecting HMIs in prepared solutions in laboratories. Only a minor study shows FET practicality of HMIs sensor in real samples. De facto, it remains a challenge to implement FET sensors to monitor HMIs in real world samples. First, real world samples such as industrial water waste, blood, tap or river water and soils are complex matrices that contain unknown elements or ion species. Unknown ions could interfere with the sensor signal. Therefore, the accuracy of the FET sensors is debatable in this case. Multiple sensing HMIs using FET array sensors might be an effective solution to address this problem. Secondly, nearly all the studies in laboratories reported the performance of the FET sensor testing in single ion HMIs buffer solutions. But in reality, the HMIs exist as metal-organic complexes. Prior to sensing with the FET sensor, real HMI analyte samples are often pre-treated (extraction, separation, dilution, or filtration). An extended study using real-world samples is necessary to evaluate the possibility of present FET designs for detection of free metal ions as well as metal-organic complex ions. Otherwise, the current sensing strategies must be reconsidered. Another solution to this challenge is to integrate the FET assay with a microfluidic system to isolate free ions from the metal-organic complexes prior to sensing. Finally, real samples are commonly associated with high ionic strength electrolytes, whereby the FET sensor will be subjected to an intrinsic issue known as the screening effect. Even though nearly all the works reviewed in this paper provide FET sensitivity data in a wide range of solution concentrations, the concentration of real sample solutions may differ from what was anticipated. Under some circumstances, especially in highly concentrated real samples, the FET assay would suffer from the screening effect, which could not yield any response in the presence of the HMIs. Another area that needs to be focused on is the durability of the FET assay when exposed to real world samples over a long period. Eventually, the FET sensing interface will be degraded. Therefore, chemical surface treatment is required to protect the transducer from fouling and corrosion, which could lead to sensor malfunction.

## Figures and Tables

**Table 1:** Limit, sources, and effects of various heavy metal ion contaminations. Reprinted from [3], Copyright 2015, with permission from Elsevier.

Metals	WHO Limit ^a^ (mgL^−1^)	Common Sources	Effects
Lead (Pb)	0.05	PVC pipes in sanitation, agriculture, recycled PVC lead paints, jewellery, lead batteries, lunch boxes, etc.	Penetrates through protective blood brain barrier (BBB) and is proving to be a risk factor for Alzheimer’s disease and senile dementia; leads also to neuro-degenerative diseases, decreases IQ, kidney damage, decreased bone growth, behavioral issues, ataxia, hyperirritability and stupor
Cadmium (Cd)	0.005	Paints, pigments, electroplated parts, batteries, plastics, synthetic rubber, photographic and engraving process, photoconductors and photovoltaic cells	Renal toxicity, hypertension, weight loss, fatigue, microcytic hypochromic anaemia, lymphocytosis, pulmonary fibrosis, atherosclerosis, peripheral neuropathy, lung cancer, osteomalacia, osteoporosis and hyperuricemia
Mercury (Hg)	0.001	Combustion of coal, municipal solid waste incineration and volcanic emissions	Impaired neurologic development, effects on digestive system, immune system, lungs, kidneys, skin and eyes, minamata, acrodynia, increases salivation, hypotonia and hypertension
Arsenic (As)	0.05	Wooden electricity poles that are treated with arsenic based preservatives, pesticides, fertilizers, release of untreated effluents, oxidation of pyrite (FeS) and arseno pyrite (FeAsS)	Causes effects on central nervous system (CNS), peripheral nervous system (PNS), cardiovascular, pulmonary diseases, gastrointestinal tract (GI), genitourinary (GU), haemopoietic, dermatologic, foetal and teratogenic diseases, anorexia, brown pigmentation, hyper-pigmentation, localized edema and skin cancer
Chromium (Cr)	0.05	Leather industry, tanning, and chrome plating industries	Reproductive toxicity, embryotoxicity, teratogenicity, mutagenicity, carcinogenicity, lung cancer, dermatitis, skin ulcers, perforation of septum and irritant contact dermatitis
Silver (Ag)	0.1	Refining of copper, gold, nickel, zinc, jewellery and electroplating industries	Argyria, gastroenteritis, neuronal disorders, mental fatigue, rheumatism, knotting of cartilage, cytopathological effects in fibroblast, keratinocytes and mast cells
Zinc (Zn)	5	Soldering, cosmetics and pigments	Respiratory disorders, metal fume fever, bronchiolar leukocytes, neuronal disorder, prostate cancer risks, macular degeneration and impotence
Copper (Cu)	1.3	Fertilizers, tanning and photovoltaic cells	Adreno-corticol hyperactivity, allergies, anaemia, alopecia, arthritis, autism, cystic fibrosis, diabetes, haemorrhaging and kidney disorders

^a^ World Health Organization (WHO) tolerable limits for heavy metal ions in water.

**Table 2:** Ag^+^ analysis of the Agzyme/SWNTs/FET in river water sample. Reproduced with permission from [120]. Copyright 2018 MDPI.

Sample	Adding Ag(I) (nM)	DNAyme/SWNTs/FET (nM)	AAS (nM)	Recovery (%)
1	-	1.23	1.17	105.12
	5	6.31		102.27
2	-	1.46	1.42	102.82
	5	6.35		98.91
3	-	0.98	1.06	92.45
	5	6.14		101.32

**Table 3:** Pb^2+^ analysis of Pbzyme/SWCNTs/FET in woodland soil and paint samples. Reprinted with permission from reference [121]. Copyright 2019 John Wiley and Sons.

Sample	Pbzyme/SWNTs/FET (nM)	AES (nM)	Relative Error (%)
1	16.39 ± 3.39	17.31	5.31
2	19.34 ± 4.11	21.32	9.28
3	16.87 ± 2.67	15.56	8.42
4	10.21 ± 2.95	11.03	7.43
5	0.78 ± 0.24	0.73	6.85
6	1.43 ± 0.41	1.32	8.33
7	2.11 ± 0.37	2.23	5.38
8	1.35 ± 0.21	1.42	4.93

**Table 4:** Recent heavy metal ions sensing strategies on the Graphene FET platform.

Surface Functionalization/Modification	Target Ions	Real Sample	Linear Range	Limit of Detection (LOD)	Reference
Self-assembled 1-octadecanethiol monolayer	Hg^2+^			4.985 nM	Zhang et al. [137]
Self-assembled monolayer Alkanethiols (1-octadecanethiol and 1-Dodecanethiol)	Hg^2+^ and Pb^2+^			4.985 nM	Afsharimani et al. [141]
Guanine-rich DNA/AuNPs	Pb^2+^			20 nM	Chee et al. [143]
G-rich DNA	Pb^2+^			163.7 ng/L	Li et al. [146]
ssDNA aptamer	Hg^2+^		0.1 nM–100 nM	40 pM	Tu et al. [148]
Aptamer (3′-amine-TTC TTT CTT CCC CTT GTT TGT-C10 carboxylic acid-5′)	Hg^2+^	mussel		10 pM	An et al. [149]
LSA Aptamer (a kind of Pb^2+^ sensitive DNAzyme)	Pb^2+^	Drinking water	4.826 nM–48.826 nM	4.826 pM	Li et al. [151]
Pb^2+-^dependent DNAzyme	Pb^2+^			0.02 nM	Wen et al. [154]
Aptamer 8-17 DNAzyme	Pb^2+^	Children blood		37.5 ng/L	Wang et al. [157]
Hg-dependent DNAzyme	Hg^2+^			1 nM	Chang et al. [158]
L-Glutathione reduce (GSH)/AuNPs	Pb^2+^		10 nM–10 μM	10 nM	Zhou et al. [162]
L-Glutathione reduce (GSH)/AuNPs	Pb^2+^			6.274 nM	Sui et al. [163]
L-Glutathione reduce (GSH)/AuNP	Pb^2+^			< 4.826 nM	Maity et al. [164]
Thioglycolic acid (TGA)/AuNPs	Hg^2+^		25 nM–14.2 µM	25 nM	Chen et al. [165]
Thiacalix[4]arene (TCA)	Cu^2+^		1 μM–1 mM	1 μM	Takagiri et al. [166]
metallothionein type II protein (MT-II)	Hg^2+^ Cd^2+^	Lake water		1 nM (Hg^2+^ & Cd^2+^)	Sudibya et al. [167]
Hg ionophore	Hg^2+^			4.985 nM	Li et al. [168]
Gold nanocluster	Hg^2+^			0.24926 nM	Ayesh et al. [169]

**Table 5:** AlGaN/GaN HEMT applications in relation to electrical detection used for heavy metals detection.

Surface Functionalization/Modification	Target Ions	Matrix	Detection Range	Limit of Detection (LOD)	Reference
Mercaptopropionic Acid (MPA) and Glutathione (GSH)	Cd^2+^			2.2685 nM	Nigam et al. [183]
2,5-dimercapto-1,3,4-thiadiazole (DMTD)	Pb^2+^	Lake water		86.87 pM	Nigam et al. [189]
molybdenum disulfide (MoS_2_)	Hg^2+^		0.4985 nM–498.5 nM	57.43 pM	Nigam et al. [192]
Ion-selective membrane (ISM)	Pb^2+^ and Hg^2+^		0.1 nM–10 µM (Pb^2+^) 10 pM–10 µM (Hg^2+^)	0.1 nM (Pb^2+^) 0.01 nM (Hg^2+^)	Chen et al. [200]
Ion-selective membrane (ISM)	Pb^2+^			0.1 nM	Chen et al. [201]
Ion-selective membrane (ISM)	Pb^2+^	Tap water	0.1 nM–10 µM	0.1 nM–10 pM	Hsieh et al. [202]
Ion-selective membrane (ISM)	Hg^2+^			0.1 pM	Sukesan et al. [203]
Ion-selective membrane (ISM)	Hg^2+^		0.1 pM–10 µM	0.1 pM	Sukesan et al. [206]
Ion-selective membrane (ISM)	Hg^2+^		10 nM–0.1 mM	<10 nM	Asadnia et al. [208]
Schiff base	Zn^2+^		1 fM - 1μM	1 fM	Gu et al. [209]
Oligonucleotide (DNA)	Hg^2+^		0.01 pM–10 nM	<0.01 pM	Cheng et al. [210]
self-assembled thioglycolic acids (TGA)	Hg^2+^			0.1 µM	Ren et al. [211]
Glutathione (GSH)	Pb^2+^		0.1 pM–10 pM	0.1 pM	Jiqiang et al. [212]
